# THE CONCISE GUIDE TO PHARMACOLOGY 2019/20: Nuclear hormone receptors

**DOI:** 10.1111/bph.14750

**Published:** 2019-11-11

**Authors:** Stephen PH Alexander, John A Cidlowski, Eamonn Kelly, Alistair Mathie, John A Peters, Emma L Veale, Jane F Armstrong, Elena Faccenda, Simon D Harding, Adam J Pawson, Joanna L Sharman, Christopher Southan, Jamie A Davies, CGTP Collaborators, Laurel Coons, Peter Fuller, Kenneth S Korach, Morag Young

**Affiliations:** ^1^ School of Life Sciences University of Nottingham Medical School Nottingham NG7 2UH UK; ^2^ Department of Health and Human Services National Institute of Environmental Health Sciences, National Institutes of Health Research Triangle Park NC 27709 USA; ^3^ School of Physiology, Pharmacology and Neuroscience University of Bristol Bristol BS8 1TD UK; ^4^ Medway School of Pharmacy The Universities of Greenwich and Kent at Medway Anson Building, Central Avenue, Chatham Maritime Chatham Kent ME4 4TB UK; ^5^ Neuroscience Division, Medical Education Institute, Ninewells Hospital and Medical School University of Dundee Dundee DD1 9SY UK; ^6^ Centre for Discovery Brain Sciences University of Edinburgh Edinburgh EH8 9XD UK; ^7^ Durham USA; ^8^ Clayton Australia; ^9^ Research Triangle Park USA; ^10^ Clayton Australia

## Abstract

The Concise Guide to PHARMACOLOGY 2019/20 is the fourth in this series of biennial publications. The Concise Guide provides concise overviews of the key properties of nearly 1800 human drug targets with an emphasis on selective pharmacology (where available), plus links to the open access knowledgebase source of drug targets and their ligands (http://www.guidetopharmacology.org), which provides more detailed views of target and ligand properties. Although the Concise Guide represents approximately 400 pages, the material presented is substantially reduced compared to information and links presented on the website. It provides a permanent, citable, point‐in‐time record that will survive database updates. The full contents of this section can be found at http://onlinelibrary.wiley.com/doi/10.1111/bph.14750. Nuclear hormone receptors are one of the six major pharmacological targets into which the Guide is divided, with the others being: G protein‐coupled receptors, catalytic receptors, enzymes and transporters. These are presented with nomenclature guidance and summary information on the best available pharmacological tools, alongside key references and suggestions for further reading. The landscape format of the Concise Guide is designed to facilitate comparison of related targets from material contemporary to mid‐2019, and supersedes data presented in the 2017/18, 2015/16 and 2013/14 Concise Guides and previous Guides to Receptors and Channels. It is produced in close conjunction with the International Union of Basic and Clinical Pharmacology Committee on Receptor Nomenclature and Drug Classification (NC‐IUPHAR), therefore, providing official IUPHAR classification and nomenclature for human drug targets, where appropriate.

1

### Conflict of interest

The authors state that there are no conflicts of interest to disclose.

### Overview

Nuclear receptors are specialised transcription factors with commonalities of sequence and structure, which bind as homo‐ or heterodimers to specific consensus sequences of DNA (response elements) in the promoter region of particular target genes. They regulate (either promoting or repressing) transcription of these target genes in response to a variety of endogenous ligands. Endogenous agonists are hydrophobic entities which, when bound to the receptor promote conformational changes in the receptor to allow recruitment (or dissociation) of protein partners, generating a large multiprotein complex.

Two major subclasses of nuclear receptors with identified endogenous agonists can be identified: steroid and non‐steroid hormone receptors. Steroid hormone receptors function typically as dimeric entities and are thought to be resident outside the nucleus in the unliganded state in a complex with chaperone proteins, which are liberated upon agonist binding. Migration to the nucleus and interaction with other regulators of gene transcription, including RNA polymerase, acetyltransferases and deacetylases, allows gene transcription to be regulated. Non‐steroid hormone receptors typically exhibit a greater distribution in the nucleus in the unliganded state and interact with other nuclear receptors to form heterodimers, as well as with other regulators of gene transcription, leading to changes in gene transcription upon agonist binding.

Selectivity of gene regulation is brought about through interaction of nuclear receptors with particular consensus sequences of DNA, which are arranged typically as repeats or inverted palindromes to allow accumulation of multiple transcription factors in the promoter regions of genes.

### Family structure

S230 1A. Thyroid hormone receptors

S231 1B. Retinoic acid receptors

S232 1C. Peroxisome proliferator‐activated receptors

S233 1D. Rev‐Erb receptors

S234 1F. Retinoic acid‐related orphans

S234 1H. Liver X receptor‐like receptors

S235 1I. Vitamin D receptor‐like receptors

S236 2A. Hepatocyte nuclear factor‐4 receptors

S237 2B. Retinoid X receptors

S238 2C. Testicular receptors

S238 2E. Tailless‐like receptors

S239 2F. COUP‐TF‐like receptors

S239 3B. Estrogen‐related receptors

S240 4A. Nerve growth factor IB‐like receptors

S241 5A. Fushi tarazu F1‐like receptors

S241 6A. Germ cell nuclear factor receptors

S242 0B. DAX‐like receptors

S242 Steroid hormone receptors

S243 3A. Estrogen receptors

S244 3C. 3‐Ketosteroid receptors

## http://www.guidetopharmacology.org/GRAC/FamilyDisplayForward?familyId=84

### Overview

Thyroid hormone receptors (**TRs, nomenclature as agreed by the NC‐IUPHAR Subcommittee on Nuclear Hormone Receptors [** http://www.ncbi.nlm.nih.gov/pubmed/17132849?dopt=AbstractPlus **]**) are nuclear hormone receptors of the NR1A family, with diverse roles regulating macronutrient metabolism, cognition and cardiovascular homeostasis. TRs are activated by thyroxine (http://www.guidetopharmacology.org/GRAC/LigandDisplayForward?ligandId=2635) and thyroid hormone (http://www.guidetopharmacology.org/GRAC/LigandDisplayForward?ligandId=2634). Once activated by a ligand, the receptor acts as a transcription factor either as a monomer, homodimer or heterodimer with members of the retinoid X receptor family. http://www.guidetopharmacology.org/GRAC/LigandDisplayForward?ligandId=2633 has been described as an antagonist at TRs with modest selectivity for TRβ [http://www.ncbi.nlm.nih.gov/pubmed/12109914?dopt=AbstractPlus].

**Table:** 

Nomenclature	http://www.guidetopharmacology.org/GRAC/ObjectDisplayForward?objectId=588	http://www.guidetopharmacology.org/GRAC/ObjectDisplayForward?objectId=589
Systematic nomenclature	NR1A1	NR1A2
HGNC, UniProt	https://www.genenames.org/data/gene‐symbol‐report/#!/hgnc_id/HGNC:11796, http://www.uniprot.org/uniprot/P10827	https://www.genenames.org/data/gene‐symbol‐report/#!/hgnc_id/HGNC:11799, http://www.uniprot.org/uniprot/P10828
Rank order of potency	http://www.guidetopharmacology.org/GRAC/LigandDisplayForward?ligandId=2634 > http://www.guidetopharmacology.org/GRAC/LigandDisplayForward?ligandId=2635	http://www.guidetopharmacology.org/GRAC/LigandDisplayForward?ligandId=2634 > http://www.guidetopharmacology.org/GRAC/LigandDisplayForward?ligandId=2635
Agonists	http://www.guidetopharmacology.org/GRAC/LigandDisplayForward?ligandId=6951 [http://www.ncbi.nlm.nih.gov/pubmed/6777394?dopt=AbstractPlus]	http://www.guidetopharmacology.org/GRAC/LigandDisplayForward?ligandId=6951 [http://www.ncbi.nlm.nih.gov/pubmed/6777394?dopt=AbstractPlus]
Selective agonists	–	http://www.guidetopharmacology.org/GRAC/LigandDisplayForward?ligandId=2639 [http://www.ncbi.nlm.nih.gov/pubmed/9653548?dopt=AbstractPlus, http://www.ncbi.nlm.nih.gov/pubmed/269396?dopt=AbstractPlus]

### Comments

An interaction with integrin αVβ3 has been suggested to underlie plasma membrane localization of TRs and non‐genomic signalling [http://www.ncbi.nlm.nih.gov/pubmed/15802494?dopt=AbstractPlus].One splice variant, TRα_2_, lacks a functional DNA‐binding domain and appears to act as a transcription suppressor.

Although radioligand binding assays have been described for these receptors, the radioligands are not commercially available.

### Further reading on 1A. Thyroid hormone receptors

Elbers LP *et al*. (2016) Thyroid Hormone Mimetics: the Past, Current Status and Future Challenges. *Curr Atheroscler Rep* **18**: 14 https://www.ncbi.nlm.nih.gov/pubmed/26886134?dopt=AbstractPlus

Flamant F *et al*. (2006) International Union of Pharmacology. LIX. The pharmacology and classification of the nuclear receptor superfamily: thyroid hormone receptors. *Pharmacol. Rev*. **58**: 705‐11 https://www.ncbi.nlm.nih.gov/pubmed/17132849?dopt=AbstractPlus

Mendoza A *et al*. (2017) New insights into thyroid hormone action. *Pharmacol. Ther*. **173**: 135‐145 https://www.ncbi.nlm.nih.gov/pubmed/28174093?dopt=AbstractPlus

## http://www.guidetopharmacology.org/GRAC/FamilyDisplayForward?familyId=85

### Overview

Retinoic acid receptors (**nomenclature as agreed by the NC‐IUPHAR Subcommittee on Nuclear Hormone Receptors [** http://www.ncbi.nlm.nih.gov/pubmed/17132850?dopt=AbstractPlus **]**) are nuclear hormone receptors of the NR1B family activated by the vitamin A‐derived agonists http://www.guidetopharmacology.org/GRAC/LigandDisplayForward?ligandId=2644 (ATRA) and http://www.guidetopharmacology.org/GRAC/LigandDisplayForward?ligandId=2645, and the RAR‐selective synthetic agonists http://www.guidetopharmacology.org/GRAC/LigandDisplayForward?ligandId=2646 and http://www.guidetopharmacology.org/GRAC/LigandDisplayForward?ligandId=5429. http://www.guidetopharmacology.org/GRAC/LigandDisplayForward?ligandId=2641 is a family‐selective antagonist [http://www.ncbi.nlm.nih.gov/pubmed/19477412?dopt=AbstractPlus].

**Table:** 

Nomenclature	http://www.guidetopharmacology.org/GRAC/ObjectDisplayForward?objectId=590	http://www.guidetopharmacology.org/GRAC/ObjectDisplayForward?objectId=591	http://www.guidetopharmacology.org/GRAC/ObjectDisplayForward?objectId=592
Systematic nomenclature	NR1B1	NR1B2	NR1B3
HGNC, UniProt	https://www.genenames.org/data/gene‐symbol‐report/#!/hgnc_id/HGNC:9864, http://www.uniprot.org/uniprot/P10276	https://www.genenames.org/data/gene‐symbol‐report/#!/hgnc_id/HGNC:9865, http://www.uniprot.org/uniprot/P10826	https://www.genenames.org/data/gene‐symbol‐report/#!/hgnc_id/HGNC:9866, http://www.uniprot.org/uniprot/P13631
Agonists	http://www.guidetopharmacology.org/GRAC/LigandDisplayForward?ligandId=2644 [http://www.ncbi.nlm.nih.gov/pubmed/19058965?dopt=AbstractPlus]	http://www.guidetopharmacology.org/GRAC/LigandDisplayForward?ligandId=2644 [http://www.ncbi.nlm.nih.gov/pubmed/19058965?dopt=AbstractPlus]	http://www.guidetopharmacology.org/GRAC/LigandDisplayForward?ligandId=2644 [http://www.ncbi.nlm.nih.gov/pubmed/19058965?dopt=AbstractPlus]
Sub/family‐selective agonists	http://www.guidetopharmacology.org/GRAC/LigandDisplayForward?ligandId=6952 [http://www.ncbi.nlm.nih.gov/pubmed/19058965?dopt=AbstractPlus]	http://www.guidetopharmacology.org/GRAC/LigandDisplayForward?ligandId=6952 [http://www.ncbi.nlm.nih.gov/pubmed/19058965?dopt=AbstractPlus], http://www.guidetopharmacology.org/GRAC/LigandDisplayForward?ligandId=5429 [http://www.ncbi.nlm.nih.gov/pubmed/8544175?dopt=AbstractPlus]	http://www.guidetopharmacology.org/GRAC/LigandDisplayForward?ligandId=6952 [http://www.ncbi.nlm.nih.gov/pubmed/19058965?dopt=AbstractPlus], http://www.guidetopharmacology.org/GRAC/LigandDisplayForward?ligandId=5429 [http://www.ncbi.nlm.nih.gov/pubmed/8544175?dopt=AbstractPlus]
Selective agonists	http://www.guidetopharmacology.org/GRAC/LigandDisplayForward?ligandId=2650 [http://www.ncbi.nlm.nih.gov/pubmed/10421757?dopt=AbstractPlus], http://www.guidetopharmacology.org/GRAC/LigandDisplayForward?ligandId=2648 [http://www.ncbi.nlm.nih.gov/pubmed/20925433?dopt=AbstractPlus], http://www.guidetopharmacology.org/GRAC/LigandDisplayForward?ligandId=2647 [http://www.ncbi.nlm.nih.gov/pubmed/1656191?dopt=AbstractPlus]	http://www.guidetopharmacology.org/GRAC/LigandDisplayForward?ligandId=4054 [http://www.ncbi.nlm.nih.gov/pubmed/16302793?dopt=AbstractPlus], http://www.guidetopharmacology.org/GRAC/LigandDisplayForward?ligandId=4055 [http://www.ncbi.nlm.nih.gov/pubmed/19239230?dopt=AbstractPlus, http://www.ncbi.nlm.nih.gov/pubmed/16302793?dopt=AbstractPlus]	http://www.guidetopharmacology.org/GRAC/LigandDisplayForward?ligandId=3431 [http://www.ncbi.nlm.nih.gov/pubmed/8544175?dopt=AbstractPlus]
Selective antagonists	http://www.guidetopharmacology.org/GRAC/LigandDisplayForward?ligandId=2642 (pIC_50_ 6.3–7.2) [http://www.ncbi.nlm.nih.gov/pubmed/1323127?dopt=AbstractPlus, http://www.ncbi.nlm.nih.gov/pubmed/8264595?dopt=AbstractPlus]	–	http://www.guidetopharmacology.org/GRAC/LigandDisplayForward?ligandId=4062 [http://www.ncbi.nlm.nih.gov/pubmed/10723137?dopt=AbstractPlus]

### Comments

http://www.guidetopharmacology.org/GRAC/LigandDisplayForward?ligandId=2642 has been suggested to be a PPARγ agonist [http://www.ncbi.nlm.nih.gov/pubmed/17290005?dopt=AbstractPlus]. http://www.guidetopharmacology.org/GRAC/LigandDisplayForward?ligandId=3433 is an antagonist with selectivity for RARα and RARβ compared with RARγ [http://www.ncbi.nlm.nih.gov/pubmed/10331664?dopt=AbstractPlus].

### Further reading on 1B. Retinoic acid receptors

Duong V *et al*. (2011) The molecular physiology of nuclear retinoic acid receptors. From health to disease. *Biochim. Biophys. Acta* **1812**: 1023‐31 [https://www.ncbi.nlm.nih.gov/pubmed/20970498?dopt=AbstractPlus]

Germain P *et al*. (2006) International Union of Pharmacology. LX. Retinoic acid receptors. *Pharmacol. Rev*. **58**: 712‐25 https://www.ncbi.nlm.nih.gov/pubmed/17132850?dopt=AbstractPlus

Larange A *et al*. (2016) Retinoic Acid and Retinoic Acid Receptors as Pleiotropic Modulators of the Immune System. *Annu. Rev. Immunol*. **34**: 369‐94 https://www.ncbi.nlm.nih.gov/pubmed/27168242?dopt=AbstractPlus

Saeed A *et al*. (2017) The interrelationship between bile acid and vitamin A homeostasis. *Biochim. Biophys. Acta* **1862**: 496‐512 https://www.ncbi.nlm.nih.gov/pubmed/28111285?dopt=AbstractPlus

## http://www.guidetopharmacology.org/GRAC/FamilyDisplayForward?familyId=86

### Overview

Peroxisome proliferator‐activated receptors (**PPARs, nomenclature as agreed by the** NC‐IUPHAR **Subcommittee on Nuclear Hormone Receptors** [http://www.ncbi.nlm.nih.gov/pubmed/17132851?dopt=AbstractPlus]) are nuclear hormone receptors of the NR1C family, with diverse roles regulating lipid homeostasis, cellular differentiation, proliferation and the immune response. PPARs have many potential endogenous agonists [http://www.ncbi.nlm.nih.gov/pubmed/12749590?dopt=AbstractPlus, http://www.ncbi.nlm.nih.gov/pubmed/17132851?dopt=AbstractPlus], including http://www.guidetopharmacology.org/GRAC/LigandDisplayForward?ligandId=1877, prostacyclin (http://www.guidetopharmacology.org/GRAC/LigandDisplayForward?ligandId=1915), many fatty acids and their oxidation products, lysophosphatidic acid (http://www.guidetopharmacology.org/GRAC/LigandDisplayForward?ligandId=2906) [http://www.ncbi.nlm.nih.gov/pubmed/12502787?dopt=AbstractPlus], http://www.guidetopharmacology.org/GRAC/LigandDisplayForward?ligandId=5426, http://www.guidetopharmacology.org/GRAC/LigandDisplayForward?ligandId=3401, http://www.guidetopharmacology.org/GRAC/LigandDisplayForward?ligandId=5427, http://www.guidetopharmacology.org/GRAC/LigandDisplayForward?ligandId=5428 and leukotriene B4 (http://www.guidetopharmacology.org/GRAC/LigandDisplayForward?ligandId=2487). http://www.guidetopharmacology.org/GRAC/LigandDisplayForward?ligandId=2668 acts as a non‐selective agonist for the PPAR family [http://www.ncbi.nlm.nih.gov/pubmed/10691680?dopt=AbstractPlus]. These receptors also bind hypolipidaemic drugs (PPARα) and anti‐diabetic thiazolidinediones (PPARγ), as well as many non‐steroidal anti‐inflammatory drugs, such as http://www.guidetopharmacology.org/GRAC/LigandDisplayForward?ligandId=5425 and http://www.guidetopharmacology.org/GRAC/LigandDisplayForward?ligandId=1909. Once activated by a ligand, the receptor forms a heterodimer with members of the retinoid X receptor family and can act as a transcription factor. Although radioligand binding assays have been described for all three receptors, the radioligands are not commercially available. Commonly, receptor occupancy studies are conducted using fluorescent ligands and truncated forms of the receptor limited to the ligand binding domain.

**Table:** 

Nomenclature	http://www.guidetopharmacology.org/GRAC/ObjectDisplayForward?objectId=593	http://www.guidetopharmacology.org/GRAC/ObjectDisplayForward?objectId=594	http://www.guidetopharmacology.org/GRAC/ObjectDisplayForward?objectId=595
Systematic nomenclature	NR1C1	NR1C2	NR1C3
HGNC, UniProt	https://www.genenames.org/data/gene‐symbol‐report/#!/hgnc_id/HGNC:9232, http://www.uniprot.org/uniprot/Q07869	https://www.genenames.org/data/gene‐symbol‐report/#!/hgnc_id/HGNC:9235, http://www.uniprot.org/uniprot/Q03181	https://www.genenames.org/data/gene‐symbol‐report/#!/hgnc_id/HGNC:9236, http://www.uniprot.org/uniprot/P37231
Selective agonists	http://www.guidetopharmacology.org/GRAC/LigandDisplayForward?ligandId=2674 [http://www.ncbi.nlm.nih.gov/pubmed/10389847?dopt=AbstractPlus, http://www.ncbi.nlm.nih.gov/pubmed/11354382?dopt=AbstractPlus], http://www.guidetopharmacology.org/GRAC/LigandDisplayForward?ligandId=6691 [http://www.ncbi.nlm.nih.gov/pubmed/18971326?dopt=AbstractPlus], http://www.guidetopharmacology.org/GRAC/LigandDisplayForward?ligandId=2666 [http://www.ncbi.nlm.nih.gov/pubmed/10691680?dopt=AbstractPlus], http://www.guidetopharmacology.org/GRAC/LigandDisplayForward?ligandId=3439 [http://www.ncbi.nlm.nih.gov/pubmed/21889235?dopt=AbstractPlus]	http://www.guidetopharmacology.org/GRAC/LigandDisplayForward?ligandId=2686 [http://www.ncbi.nlm.nih.gov/pubmed/15939051?dopt=AbstractPlus, http://www.ncbi.nlm.nih.gov/pubmed/12699745?dopt=AbstractPlus], http://www.guidetopharmacology.org/GRAC/LigandDisplayForward?ligandId=2687 [http://www.ncbi.nlm.nih.gov/pubmed/11309497?dopt=AbstractPlus]	http://www.guidetopharmacology.org/GRAC/LigandDisplayForward?ligandId=2703 [http://www.ncbi.nlm.nih.gov/pubmed/10389847?dopt=AbstractPlus], http://www.guidetopharmacology.org/GRAC/LigandDisplayForward?ligandId=2717 (Partial agonist) [http://www.ncbi.nlm.nih.gov/pubmed/11043571?dopt=AbstractPlus], http://www.guidetopharmacology.org/GRAC/LigandDisplayForward?ligandId=1056 [http://www.ncbi.nlm.nih.gov/pubmed/9836620?dopt=AbstractPlus, http://www.ncbi.nlm.nih.gov/pubmed/9013583?dopt=AbstractPlus, http://www.ncbi.nlm.nih.gov/pubmed/9454824?dopt=AbstractPlus], http://www.guidetopharmacology.org/GRAC/LigandDisplayForward?ligandId=2693 [http://www.ncbi.nlm.nih.gov/pubmed/9836620?dopt=AbstractPlus, http://www.ncbi.nlm.nih.gov/pubmed/9454824?dopt=AbstractPlus], http://www.guidetopharmacology.org/GRAC/LigandDisplayForward?ligandId=2694 [http://www.ncbi.nlm.nih.gov/pubmed/9836620?dopt=AbstractPlus, http://www.ncbi.nlm.nih.gov/pubmed/11095972?dopt=AbstractPlus, http://www.ncbi.nlm.nih.gov/pubmed/9454824?dopt=AbstractPlus], http://www.guidetopharmacology.org/GRAC/LigandDisplayForward?ligandId=2711 [http://www.ncbi.nlm.nih.gov/pubmed/9836620?dopt=AbstractPlus]
Selective antagonists	http://www.guidetopharmacology.org/GRAC/LigandDisplayForward?ligandId=3440 (pIC_50_ 6.6) [http://www.ncbi.nlm.nih.gov/pubmed/11845213?dopt=AbstractPlus]	http://www.guidetopharmacology.org/GRAC/LigandDisplayForward?ligandId=3441 (pIC_50_ 6.5) [http://www.ncbi.nlm.nih.gov/pubmed/17975020?dopt=AbstractPlus]	http://www.guidetopharmacology.org/GRAC/LigandDisplayForward?ligandId=3444 (p*K*_i_ 9) [http://www.ncbi.nlm.nih.gov/pubmed/11877444?dopt=AbstractPlus], http://www.guidetopharmacology.org/GRAC/LigandDisplayForward?ligandId=3442 (Irreversible inhibition) (pIC_50_ 8.1) [http://www.ncbi.nlm.nih.gov/pubmed/12022867?dopt=AbstractPlus], http://www.guidetopharmacology.org/GRAC/LigandDisplayForward?ligandId=3443 (p*K*_i_ 6.9) [http://www.ncbi.nlm.nih.gov/pubmed/11043571?dopt=AbstractPlus]

### Comments

As with the estrogen receptor antagonists, many agents show tissue‐selective efficacy (*e.g*. [http://www.ncbi.nlm.nih.gov/pubmed/11030710?dopt=AbstractPlus, http://www.ncbi.nlm.nih.gov/pubmed/11991651?dopt=AbstractPlus, http://www.ncbi.nlm.nih.gov/pubmed/11684010?dopt=AbstractPlus]). Agonists with mixed activity at PPARα and PPARγ have also been described (*e.g* [http://www.ncbi.nlm.nih.gov/pubmed/15120604?dopt=AbstractPlus, http://www.ncbi.nlm.nih.gov/pubmed/14701675?dopt=AbstractPlus, http://www.ncbi.nlm.nih.gov/pubmed/15115385?dopt=AbstractPlus]).

### Further reading on 1C. Peroxisome proliferator‐activated receptors

Cheang WS *et al*. (2015) The peroxisome proliferator‐activated receptors in cardiovascular diseases: experimental benefits and clinical challenges. *Br. J. Pharmacol*. **172**: 5512‐22 [https://www.ncbi.nlm.nih.gov/pubmed/25438608?dopt=AbstractPlus]

Gross B *et al*. (2017) PPARs in obesity‐induced T2DM, dyslipidaemia and NAFLD. *Nat Rev Endocrinol* **13**: 36‐49 [https://www.ncbi.nlm.nih.gov/pubmed/27636730?dopt=AbstractPlus]

Hallenborg P *et al*. (2016) The elusive endogenous adipogenic PPARγ agonists: Lining up the suspects. *Prog. Lipid Res*. **61**: 149‐62 [https://www.ncbi.nlm.nih.gov/pubmed/26703188?dopt=AbstractPlus]

Michalik L *et al*. (2006) International Union of Pharmacology. LXI. Peroxisome proliferator‐activated receptors. *Pharmacol. Rev*. **58**: 726‐41 [https://www.ncbi.nlm.nih.gov/pubmed/17132851?dopt=AbstractPlus]

Sauer S. (2015) Ligands for the Nuclear Peroxisome Proliferator‐Activated Receptor Gamma. *Trends Pharmacol. Sci*. **36**: 688‐704 [https://www.ncbi.nlm.nih.gov/pubmed/26435213?dopt=AbstractPlus]

## http://www.guidetopharmacology.org/GRAC/FamilyDisplayForward?familyId=87

### Overview

Rev‐erb receptors (**nomenclature as agreed by the** NC‐IUPHAR **Subcommittee on Nuclear Hormone Receptors** [http://www.ncbi.nlm.nih.gov/pubmed/17132856?dopt=AbstractPlus]) have yet to be officially paired with an endogenous ligand, but are thought to be activated by heme.

**Table:** 

Nomenclature	http://www.guidetopharmacology.org/GRAC/ObjectDisplayForward?objectId=596	http://www.guidetopharmacology.org/GRAC/ObjectDisplayForward?objectId=597
Systematic nomenclature	NR1D1	NR1D2
HGNC, UniProt	https://www.genenames.org/data/gene‐symbol‐report/#!/hgnc_id/HGNC:7962, http://www.uniprot.org/uniprot/P20393	https://www.genenames.org/data/gene‐symbol‐report/#!/hgnc_id/HGNC:7963, http://www.uniprot.org/uniprot/Q14995
Endogenous agonists	http://www.guidetopharmacology.org/GRAC/LigandDisplayForward?ligandId=4349 [http://www.ncbi.nlm.nih.gov/pubmed/18037887?dopt=AbstractPlus, http://www.ncbi.nlm.nih.gov/pubmed/18006707?dopt=AbstractPlus]	http://www.guidetopharmacology.org/GRAC/LigandDisplayForward?ligandId=4349 [http://www.ncbi.nlm.nih.gov/pubmed/24872411?dopt=AbstractPlus, http://www.ncbi.nlm.nih.gov/pubmed/18037887?dopt=AbstractPlus, http://www.ncbi.nlm.nih.gov/pubmed/18006707?dopt=AbstractPlus]
Selective agonists	http://www.guidetopharmacology.org/GRAC/LigandDisplayForward?ligandId=2903 [http://www.ncbi.nlm.nih.gov/pubmed/20677822?dopt=AbstractPlus], http://www.guidetopharmacology.org/GRAC/LigandDisplayForward?ligandId=2903 [http://www.ncbi.nlm.nih.gov/pubmed/21043485?dopt=AbstractPlus]	–
Selective antagonists	http://www.guidetopharmacology.org/GRAC/LigandDisplayForward?ligandId=2904 (pIC_50_ 6.5) [http://www.ncbi.nlm.nih.gov/pubmed/21043485?dopt=AbstractPlus]	–

### Further reading on 1D. Rev‐Erb receptors

Benoit G *et al*. (2006) International Union of Pharmacology. LXVI. Orphan nuclear receptors. *Pharmacol. Rev*. **58**: 798‐836 [https://www.ncbi.nlm.nih.gov/pubmed/17132856?dopt=AbstractPlus]

Gonzalez‐Sanchez E *et al*. (2015) Nuclear receptors in acute and chronic cholestasis. *Dig Dis* **33**: 357‐66 [https://www.ncbi.nlm.nih.gov/pubmed/26045270?dopt=AbstractPlus]

Gustafson CL *et al*. (2015) Emerging models for the molecular basis of mammalian circadian timing. *Biochemistry* **54**: 134‐49 [https://www.ncbi.nlm.nih.gov/pubmed/25303119?dopt=AbstractPlus]

Sousa EH *et al*. (2017) Drug discovery targeting heme‐based sensors and their coupled activities. *J. Inorg. Biochem*. **167**: 12‐20 [https://www.ncbi.nlm.nih.gov/pubmed/27893989?dopt=AbstractPlus]

## http://www.guidetopharmacology.org/GRAC/FamilyDisplayForward?familyId=88

### Overview

Retinoic acid receptor‐related orphan receptors (ROR, **nomenclature as agreed by the** NC‐IUPHAR **Subcommittee on Nuclear Hormone Receptors** [http://www.ncbi.nlm.nih.gov/pubmed/17132856?dopt=AbstractPlus]) have yet to be assigned a definitive endogenous ligand, although RORα may be synthesized with a ‘captured’ agonist such as http://www.guidetopharmacology.org/GRAC/LigandDisplayForward?ligandId=2718 [http://www.ncbi.nlm.nih.gov/pubmed/14722075?dopt=AbstractPlus, http://www.ncbi.nlm.nih.gov/pubmed/12467577?dopt=AbstractPlus].

**Table:** 

Nomenclature	http://www.guidetopharmacology.org/GRAC/ObjectDisplayForward?objectId=598	http://www.guidetopharmacology.org/GRAC/ObjectDisplayForward?objectId=599	http://www.guidetopharmacology.org/GRAC/ObjectDisplayForward?objectId=600
Systematic nomenclature	NR1F1	NR1F2	NR1F3
HGNC, UniProt	https://www.genenames.org/data/gene‐symbol‐report/#!/hgnc_id/HGNC:10258, http://www.uniprot.org/uniprot/P35398	https://www.genenames.org/data/gene‐symbol‐report/#!/hgnc_id/HGNC:10259, http://www.uniprot.org/uniprot/Q92753	https://www.genenames.org/data/gene‐symbol‐report/#!/hgnc_id/HGNC:10260, http://www.uniprot.org/uniprot/P51449
Endogenous agonists	http://www.guidetopharmacology.org/GRAC/LigandDisplayForward?ligandId=2718 [http://www.ncbi.nlm.nih.gov/pubmed/12467577?dopt=AbstractPlus, http://www.ncbi.nlm.nih.gov/pubmed/8858107?dopt=AbstractPlus]	–	–
Selective agonists	http://www.guidetopharmacology.org/GRAC/LigandDisplayForward?ligandId=3432 [http://www.ncbi.nlm.nih.gov/pubmed/14622968?dopt=AbstractPlus], http://www.guidetopharmacology.org/GRAC/LigandDisplayForward?ligandId=2738 [http://www.ncbi.nlm.nih.gov/pubmed/14622968?dopt=AbstractPlus, http://www.ncbi.nlm.nih.gov/pubmed/12467577?dopt=AbstractPlus]	–	–
Comments	–	–	The immune system function of RORC proteins most likely resides with expression of the RORγt isoform by immature CD4^+^/CD8^+^ cells in the thymus [http://www.ncbi.nlm.nih.gov/pubmed/15247480?dopt=AbstractPlus, http://www.ncbi.nlm.nih.gov/pubmed/10875923?dopt=AbstractPlus] and in lymphoid tissue inducer (LTi) cells [http://www.ncbi.nlm.nih.gov/pubmed/14691482?dopt=AbstractPlus].

### Comments

http://www.guidetopharmacology.org/GRAC/LigandDisplayForward?ligandId=2644 shows selectivity for RORβ within the ROR family [http://www.ncbi.nlm.nih.gov/pubmed/12958591?dopt=AbstractPlus]. RORα has been suggested to be a nuclear receptor responding to http://www.guidetopharmacology.org/GRAC/LigandDisplayForward?ligandId=224 [http://www.ncbi.nlm.nih.gov/pubmed/7885826?dopt=AbstractPlus].

### Further reading on 1F. Retinoic acid‐related orphans

Benoit G *et al*. (2006) International Union of Pharmacology. LXVI. Orphan nuclear receptors. *Pharmacol. Rev*. **58**: 798‐836 [https://www.ncbi.nlm.nih.gov/pubmed/17132856?dopt=AbstractPlus]

Cyr P *et al*. (2016) Recent progress on nuclear receptor RORγ modulators. *Bioorg. Med. Chem. Lett*. **26**: 4387‐4393 [https://www.ncbi.nlm.nih.gov/pubmed/27542308?dopt=AbstractPlus]

Germain P *et al*. (2006) Overview of nomenclature of nuclear receptors. *Pharmacol. Rev*. **58**: 685‐704 [https://www.ncbi.nlm.nih.gov/pubmed/17132848?dopt=AbstractPlus]

Guillemot‐Legris O *et al*. (2016) Oxysterols in Metabolic Syndrome: From Bystander Molecules to Bioactive Lipids. *Trends Mol Med* **22**: 594‐614 [https://www.ncbi.nlm.nih.gov/pubmed/27286741?dopt=AbstractPlus]

Mutemberezi V *et al*. (2016) Oxysterols: From cholesterol metabolites to key mediators. *Prog. Lipid Res*. **64**: 152‐169 [https://www.ncbi.nlm.nih.gov/pubmed/27687912?dopt=AbstractPlus]

## http://www.guidetopharmacology.org/GRAC/FamilyDisplayForward?familyId=89

### Overview

Liver X and farnesoid X receptors (LXR and FXR, **nomenclature as agreed by the** NC‐IUPHAR **Subcommittee on Nuclear Hormone Receptors** [http://www.ncbi.nlm.nih.gov/pubmed/17132852?dopt=AbstractPlus]) are members of a steroid analogue‐activated nuclear receptor subfamily, which form heterodimers with members of the retinoid X receptor family. Endogenous ligands for LXRs include hydroxycholesterols (OHC), while FXRs appear to be activated by bile acids. In humans and primates, *NR1H5P* is a pseudogene. However, in other mammals, it encodes a functional nuclear hormone receptor that appears to be involved in cholesterol biosynthesis [http://www.ncbi.nlm.nih.gov/pubmed/12529392?dopt=AbstractPlus].

**Table:** 

Nomenclature	http://www.guidetopharmacology.org/GRAC/ObjectDisplayForward?objectId=603	http://www.guidetopharmacology.org/GRAC/ObjectDisplayForward?objectId=604	http://www.guidetopharmacology.org/GRAC/ObjectDisplayForward?objectId=602	http://www.guidetopharmacology.org/GRAC/ObjectDisplayForward?objectId=601
Systematic nomenclature	NR1H4	NR1H5	NR1H3	NR1H2
HGNC, UniProt	https://www.genenames.org/data/gene‐symbol‐report/#!/hgnc_id/HGNC:7967, http://www.uniprot.org/uniprot/Q96RI1	https://www.genenames.org/data/gene‐symbol‐report/#!/hgnc_id/HGNC:32673, –	https://www.genenames.org/data/gene‐symbol‐report/#!/hgnc_id/HGNC:7966, http://www.uniprot.org/uniprot/Q13133	https://www.genenames.org/data/gene‐symbol‐report/#!/hgnc_id/HGNC:7965, http://www.uniprot.org/uniprot/P55055
Potency order	http://www.guidetopharmacology.org/GRAC/LigandDisplayForward?ligandId=608 > http://www.guidetopharmacology.org/GRAC/LigandDisplayForward?ligandId=611, http://www.guidetopharmacology.org/GRAC/LigandDisplayForward?ligandId=610 [http://www.ncbi.nlm.nih.gov/pubmed/10334992?dopt=AbstractPlus, http://www.ncbi.nlm.nih.gov/pubmed/10334993?dopt=AbstractPlus]	–	http://www.guidetopharmacology.org/GRAC/LigandDisplayForward?ligandId=3434, http://www.guidetopharmacology.org/GRAC/LigandDisplayForward?ligandId=2742, http://www.guidetopharmacology.org/GRAC/LigandDisplayForward?ligandId=2750 > http://www.guidetopharmacology.org/GRAC/LigandDisplayForward?ligandId=2885, http://www.guidetopharmacology.org/GRAC/LigandDisplayForward?ligandId=2752 [http://www.ncbi.nlm.nih.gov/pubmed/9013544?dopt=AbstractPlus]	http://www.guidetopharmacology.org/GRAC/LigandDisplayForward?ligandId=3434, http://www.guidetopharmacology.org/GRAC/LigandDisplayForward?ligandId=2742, http://www.guidetopharmacology.org/GRAC/LigandDisplayForward?ligandId=2750 > http://www.guidetopharmacology.org/GRAC/LigandDisplayForward?ligandId=2885, http://www.guidetopharmacology.org/GRAC/LigandDisplayForward?ligandId=2752 [http://www.ncbi.nlm.nih.gov/pubmed/9013544?dopt=AbstractPlus]
Endogenous agonists	–	http://www.guidetopharmacology.org/GRAC/LigandDisplayForward?ligandId=2746 [http://www.ncbi.nlm.nih.gov/pubmed/12529392?dopt=AbstractPlus] – Mouse	–	–
Selective agonists	http://www.guidetopharmacology.org/GRAC/LigandDisplayForward?ligandId=2743 [http://www.ncbi.nlm.nih.gov/pubmed/10956205?dopt=AbstractPlus], http://www.guidetopharmacology.org/GRAC/LigandDisplayForward?ligandId=3435 [http://www.ncbi.nlm.nih.gov/pubmed/12166927?dopt=AbstractPlus], http://www.guidetopharmacology.org/GRAC/LigandDisplayForward?ligandId=2744 [http://www.ncbi.nlm.nih.gov/pubmed/12718892?dopt=AbstractPlus]	–	–	–
Selective antagonists	http://www.guidetopharmacology.org/GRAC/LigandDisplayForward?ligandId=2745 (pIC_50_ 5.7–6) [http://www.ncbi.nlm.nih.gov/pubmed/12089353?dopt=AbstractPlus]	–	–	–

### Comments

http://www.guidetopharmacology.org/GRAC/LigandDisplayForward?ligandId=2755 [http://www.ncbi.nlm.nih.gov/pubmed/10968783?dopt=AbstractPlus] and http://www.guidetopharmacology.org/GRAC/LigandDisplayForward?ligandId=2754 [http://www.ncbi.nlm.nih.gov/pubmed/11985463?dopt=AbstractPlus] are synthetic agonists acting at both LXRα and LXRβ with less than 10‐fold selectivity.

### Further reading on 1H. Liver X receptor‐like receptors

Courtney R *et al*. (2016) LXR Regulation of Brain Cholesterol: From Development to Disease. *Trends Endocrinol. Metab*. **27**: 404‐414 [https://www.ncbi.nlm.nih.gov/pubmed/27113081?dopt=AbstractPlus]

El‐Gendy BEM *et al*. (2018) Recent Advances in the Medicinal Chemistry of Liver X Receptors. *J. Med. Chem*. **61**: 10935‐10956 [https://www.ncbi.nlm.nih.gov/pubmed/30004226?dopt=AbstractPlus]

Gadaleta RM *et al*. (2010) Bile acids and their nuclear receptor FXR: Relevance for hepatobiliary and gastrointestinal disease. *Biochim. Biophys. Acta* **1801**: 683‐92 [https://www.ncbi.nlm.nih.gov/pubmed/20399894?dopt=AbstractPlus]

Merlen G *et al*. (2017) Bile acids and their receptors during liver regeneration: “Dangerous protectors”. *Mol. Aspects Med*. **56**: 25‐33 [https://www.ncbi.nlm.nih.gov/pubmed/28302491?dopt=AbstractPlus]

Moore DD *et al*. (2006) International Union of Pharmacology. LXII. The NR1H and NR1I receptors: constitutive androstane receptor, pregnene X receptor, farnesoid X receptor alpha, farnesoid X receptor beta, liver X receptor alpha, liver X receptor beta, and vitamin D receptor. *Pharmacol. Rev*. **58**: 742‐59 [https://www.ncbi.nlm.nih.gov/pubmed/17132852?dopt=AbstractPlus]

Mouzat K *et al*. (2016) Liver X receptors: from cholesterol regulation to neuroprotection‐a new barrier against neurodegeneration in amyotrophic lateral sclerosis? *Cell. Mol. Life Sci*. **73**: 3801‐8 [https://www.ncbi.nlm.nih.gov/pubmed/27510420?dopt=AbstractPlus]

Schulman IG. (2017) Liver X receptors link lipid metabolism and inflammation. *FEBS Lett*. **591**: 2978‐2991 [https://www.ncbi.nlm.nih.gov/pubmed/28555747?dopt=AbstractPlus]

## http://www.guidetopharmacology.org/GRAC/FamilyDisplayForward?familyId=90

### Overview

Vitamin D (VDR), Pregnane X (PXR) and Constitutive Androstane (CAR) receptors (**nomenclature as agreed by the NC‐IUPHAR Subcommittee on Nuclear Hormone Receptors [** http://www.ncbi.nlm.nih.gov/pubmed/17132852?dopt=AbstractPlus **]**) are members of the NR1I family of nuclear receptors, which form heterodimers with members of the retinoid X receptor family. PXR and CAR are activated by a range of exogenous compounds, with no established endogenous physiological agonists, although high concentrations of bile acids and bile pigments activate PXR and CAR [http://www.ncbi.nlm.nih.gov/pubmed/17132852?dopt=AbstractPlus].

**Table:** 

Nomenclature	http://www.guidetopharmacology.org/GRAC/ObjectDisplayForward?objectId=605	http://www.guidetopharmacology.org/GRAC/ObjectDisplayForward?objectId=606	http://www.guidetopharmacology.org/GRAC/ObjectDisplayForward?objectId=607
Systematic nomenclature	NR1I1	NR1I2	NR1I3
HGNC, UniProt	https://www.genenames.org/data/gene‐symbol‐report/#!/hgnc_id/HGNC:12679, http://www.uniprot.org/uniprot/P11473	https://www.genenames.org/data/gene‐symbol‐report/#!/hgnc_id/HGNC:7968, http://www.uniprot.org/uniprot/O75469	https://www.genenames.org/data/gene‐symbol‐report/#!/hgnc_id/HGNC:7969, http://www.uniprot.org/uniprot/Q14994
Endogenous agonists	http://www.guidetopharmacology.org/GRAC/LigandDisplayForward?ligandId=2779 [http://www.ncbi.nlm.nih.gov/pubmed/7976510?dopt=AbstractPlus, http://www.ncbi.nlm.nih.gov/pubmed/12089348?dopt=AbstractPlus]	http://www.guidetopharmacology.org/GRAC/LigandDisplayForward?ligandId=1013 [http://www.ncbi.nlm.nih.gov/pubmed/10628745?dopt=AbstractPlus]	–
Selective agonists	http://www.guidetopharmacology.org/GRAC/LigandDisplayForward?ligandId=2777 [http://www.ncbi.nlm.nih.gov/pubmed/1472092?dopt=AbstractPlus, http://www.ncbi.nlm.nih.gov/pubmed/8573413?dopt=AbstractPlus], http://www.guidetopharmacology.org/GRAC/LigandDisplayForward?ligandId=2790	http://www.guidetopharmacology.org/GRAC/LigandDisplayForward?ligandId=2764 [http://www.ncbi.nlm.nih.gov/pubmed/10852961?dopt=AbstractPlus, http://www.ncbi.nlm.nih.gov/pubmed/10974665?dopt=AbstractPlus], http://www.guidetopharmacology.org/GRAC/LigandDisplayForward?ligandId=2759 [http://www.ncbi.nlm.nih.gov/pubmed/10628745?dopt=AbstractPlus], http://www.guidetopharmacology.org/GRAC/LigandDisplayForward?ligandId=2739 [http://www.ncbi.nlm.nih.gov/pubmed/9727070?dopt=AbstractPlus], http://www.guidetopharmacology.org/GRAC/LigandDisplayForward?ligandId=2765 [http://www.ncbi.nlm.nih.gov/pubmed/9784494?dopt=AbstractPlus, http://www.ncbi.nlm.nih.gov/pubmed/9727070?dopt=AbstractPlus]	http://www.guidetopharmacology.org/GRAC/LigandDisplayForward?ligandId=2756 [http://www.ncbi.nlm.nih.gov/pubmed/10757780?dopt=AbstractPlus] – Mouse, http://www.guidetopharmacology.org/GRAC/LigandDisplayForward?ligandId=2758 [http://www.ncbi.nlm.nih.gov/pubmed/12611900?dopt=AbstractPlus]
Selective antagonists	http://www.guidetopharmacology.org/GRAC/LigandDisplayForward?ligandId=2788 (pIC_50_ 8.2) [http://www.ncbi.nlm.nih.gov/pubmed/17125259?dopt=AbstractPlus] – Chicken, http://www.guidetopharmacology.org/GRAC/LigandDisplayForward?ligandId=2789 (pIC^50^ 7.5) [http://www.ncbi.nlm.nih.gov/pubmed/12901907?dopt=AbstractPlus, http://www.ncbi.nlm.nih.gov/pubmed/11094341?dopt=AbstractPlus]	–	–
Comments	–	–	http://www.guidetopharmacology.org/GRAC/LigandDisplayForward?ligandId=2330 [http://www.ncbi.nlm.nih.gov/pubmed/10748001?dopt=AbstractPlus] and http://www.guidetopharmacology.org/GRAC/LigandDisplayForward?ligandId=2755 [http://www.ncbi.nlm.nih.gov/pubmed/23665929?dopt=AbstractPlus] although acting at other sites, function as antagonists of the constitutive androstane receptor.

### Further reading on 1I. Vitamin D receptor‐like receptors

Benoit G *et al*. (2006) International Union of Pharmacology. LXVI. Orphan nuclear receptors. *Pharmacol. Rev*. **58**: 798‐836 https://www.ncbi.nlm.nih.gov/pubmed/17132856?dopt=AbstractPlus

Long MD *et al*. (2015) Vitamin D receptor and RXR in the post‐genomic era. *J. Cell. Physiol*. **230**: 758‐66 https://www.ncbi.nlm.nih.gov/pubmed/25335912?dopt=AbstractPlus

Moore DD *et al*. (2006) International Union of Pharmacology. LXII. The NR1H and NR1I receptors: constitutive androstane receptor, pregnene X receptor, farnesoid X receptor alpha, farnesoid X receptor beta, liver X receptor alpha, liver X receptor beta, and vitamin D receptor. *Pharmacol. Rev*. **58**: 742‐59 https://www.ncbi.nlm.nih.gov/pubmed/17132852?dopt=AbstractPlus

## http://www.guidetopharmacology.org/GRAC/FamilyDisplayForward?familyId=91

### Overview

The nomenclature of hepatocyte nuclear factor‐4 receptors is agreed by the **NC‐IUPHAR Subcommittee on Nuclear Hormone Receptors [** http://www.ncbi.nlm.nih.gov/pubmed/17132856?dopt=AbstractPlus **]**. While linoleic acid has been identified as the endogenous ligand for HNF4α its function remains ambiguous [http://www.ncbi.nlm.nih.gov/pubmed/19440305?dopt=AbstractPlus]. HNF4γ has yet to be paired with an endogenous ligand.

**Table:** 

Nomenclature	http://www.guidetopharmacology.org/GRAC/ObjectDisplayForward?objectId=608	http://www.guidetopharmacology.org/GRAC/ObjectDisplayForward?objectId=609
Systematic nomenclature	NR2A1	NR2A2
HGNC, UniProt	https://www.genenames.org/data/gene‐symbol‐report/#!/hgnc_id/HGNC:5024, http://www.uniprot.org/uniprot/P41235	https://www.genenames.org/data/gene‐symbol‐report/#!/hgnc_id/HGNC:5026, http://www.uniprot.org/uniprot/Q14541
Endogenous agonists	http://www.guidetopharmacology.org/GRAC/LigandDisplayForward?ligandId=1052 [http://www.ncbi.nlm.nih.gov/pubmed/19440305?dopt=AbstractPlus]	–
Selective antagonists	http://www.guidetopharmacology.org/GRAC/LigandDisplayForward?ligandId=6695 [http://www.ncbi.nlm.nih.gov/pubmed/22840769?dopt=AbstractPlus]	–
Comments	HNF4α has constitutive transactivation activity [http://www.ncbi.nlm.nih.gov/pubmed/19440305?dopt=AbstractPlus] and binds DNA as a homodimer [http://www.ncbi.nlm.nih.gov/pubmed/7651430?dopt=AbstractPlus].	–

### Further reading on 2A. Hepatocyte nuclear factor‐4 receptors

Benoit G *et al*. (2006) International Union of Pharmacology. LXVI. Orphan nuclear receptors. *Pharmacol. Rev*. **58**: 798‐836 https://www.ncbi.nlm.nih.gov/pubmed/17132856?dopt=AbstractPlus

Garattini E *et al*. (2016) Lipid‐sensors, enigmatic‐orphan and orphan nuclear receptors as therapeutic targets in breast‐cancer. *Oncotarget* **7**: 42661‐42682 https://www.ncbi.nlm.nih.gov/pubmed/26894976?dopt=AbstractPlus

Germain P *et al*. (2006) Overview of nomenclature of nuclear receptors. *Pharmacol. Rev*. **58**: 685‐704 https://www.ncbi.nlm.nih.gov/pubmed/17132848?dopt=AbstractPlus

Lu H. (2016) Crosstalk of HNF4α with extracellular and intracellular signaling pathways in the regulation of hepatic metabolism of drugs and lipids. *Acta Pharm Sin B* **6**: 393‐408 https://www.ncbi.nlm.nih.gov/pubmed/27709008?dopt=AbstractPlus

Walesky C *et al*. (2015) Role of hepatocyte nuclear factor 4α (HNF4α) in cell proliferation and cancer. *Gene Expr*. **16**: 101‐8 https://www.ncbi.nlm.nih.gov/pubmed/25700366?dopt=AbstractPlus

## http://www.guidetopharmacology.org/GRAC/FamilyDisplayForward?familyId=92

### Overview

Retinoid X receptors (**nomenclature as agreed by the NC‐IUPHAR Subcommittee on Nuclear Hormone Receptors [** http://www.ncbi.nlm.nih.gov/pubmed/17132853?dopt=AbstractPlus **]**) are NR2B family members activated by http://www.guidetopharmacology.org/GRAC/LigandDisplayForward?ligandId=2645 and the RXR‐selective agonists http://www.guidetopharmacology.org/GRAC/LigandDisplayForward?ligandId=2807 and http://www.guidetopharmacology.org/GRAC/LigandDisplayForward?ligandId=2808, sometimes referred to as rexinoids. http://www.guidetopharmacology.org/GRAC/LigandDisplayForward?ligandId=2816 [http://www.ncbi.nlm.nih.gov/pubmed/17947383?dopt=AbstractPlus] and http://www.guidetopharmacology.org/GRAC/LigandDisplayForward?ligandId=8079 [http://www.ncbi.nlm.nih.gov/pubmed/10748721?dopt=AbstractPlus] have been described as a pan‐RXR antagonists. These receptors form RXR‐RAR heterodimers and RXR‐RXR homodimers [http://www.ncbi.nlm.nih.gov/pubmed/8801176?dopt=AbstractPlus, http://www.ncbi.nlm.nih.gov/pubmed/8521508?dopt=AbstractPlus].

**Table:** 

Nomenclature	http://www.guidetopharmacology.org/GRAC/ObjectDisplayForward?objectId=610	http://www.guidetopharmacology.org/GRAC/ObjectDisplayForward?objectId=611	http://www.guidetopharmacology.org/GRAC/ObjectDisplayForward?objectId=612
Systematic nomenclature	NR2B1	NR2B2	NR2B3
HGNC, UniProt	https://www.genenames.org/data/gene‐symbol‐report/#!/hgnc_id/HGNC:10477, http://www.uniprot.org/uniprot/P19793	https://www.genenames.org/data/gene‐symbol‐report/#!/hgnc_id/HGNC:10478, http://www.uniprot.org/uniprot/P28702	https://www.genenames.org/data/gene‐symbol‐report/#!/hgnc_id/HGNC:10479, http://www.uniprot.org/uniprot/P48443
Sub/family‐selective agonists	http://www.guidetopharmacology.org/GRAC/LigandDisplayForward?ligandId=2807 [http://www.ncbi.nlm.nih.gov/pubmed/8071941?dopt=AbstractPlus, http://www.ncbi.nlm.nih.gov/pubmed/10052980?dopt=AbstractPlus, http://www.ncbi.nlm.nih.gov/pubmed/10637371?dopt=AbstractPlus]	http://www.guidetopharmacology.org/GRAC/LigandDisplayForward?ligandId=2807 [http://www.ncbi.nlm.nih.gov/pubmed/8071941?dopt=AbstractPlus, http://www.ncbi.nlm.nih.gov/pubmed/10052980?dopt=AbstractPlus, http://www.ncbi.nlm.nih.gov/pubmed/10637371?dopt=AbstractPlus]	http://www.guidetopharmacology.org/GRAC/LigandDisplayForward?ligandId=2807 [http://www.ncbi.nlm.nih.gov/pubmed/8071941?dopt=AbstractPlus, http://www.ncbi.nlm.nih.gov/pubmed/10052980?dopt=AbstractPlus, http://www.ncbi.nlm.nih.gov/pubmed/10637371?dopt=AbstractPlus]
Selective agonists	http://www.guidetopharmacology.org/GRAC/LigandDisplayForward?ligandId=2810 [http://www.ncbi.nlm.nih.gov/pubmed/11805839?dopt=AbstractPlus]	–	–

### Further reading on 2B. Retinoid X receptors

Germain P *et al*. (2006) International Union of Pharmacology. LXIII. Retinoid X receptors. *Pharmacol. Rev*. **58**: 760‐72 https://www.ncbi.nlm.nih.gov/pubmed/17132853?dopt=AbstractPlus

Long MD *et al*. (2015) Vitamin D receptor and RXR in the post‐genomic era. *J. Cell. Physiol*. **230**: 758‐66 https://www.ncbi.nlm.nih.gov/pubmed/25335912?dopt=AbstractPlus

Menéndez‐Gutiérrez MP *et al*. (2017) The multi‐faceted role of retinoid X receptor in bone remodeling. *Cell. Mol. Life Sci*. **74**: 2135‐2149 https://www.ncbi.nlm.nih.gov/pubmed/28105491?dopt=AbstractPlus

## http://www.guidetopharmacology.org/GRAC/FamilyDisplayForward?familyId=93

### Overview

Testicular receptors (**nomenclature as agreed by the NC‐IUPHAR Subcommittee on Nuclear Hormone Receptors [** http://www.ncbi.nlm.nih.gov/pubmed/17132856?dopt=AbstractPlus **]**) have yet to be officially paired with an endogenous ligand, although testicular receptor 4 has been reported to respond to retinoids.

**Table:** 

Nomenclature	http://www.guidetopharmacology.org/GRAC/ObjectDisplayForward?objectId=613	http://www.guidetopharmacology.org/GRAC/ObjectDisplayForward?objectId=614
Systematic nomenclature	NR2C1	NR2C2
HGNC, UniProt	https://www.genenames.org/data/gene‐symbol‐report/#!/hgnc_id/HGNC:7971, http://www.uniprot.org/uniprot/P13056	https://www.genenames.org/data/gene‐symbol‐report/#!/hgnc_id/HGNC:7972, http://www.uniprot.org/uniprot/P49116
Endogenous agonists	–	http://www.guidetopharmacology.org/GRAC/LigandDisplayForward?ligandId=4053 [http://www.ncbi.nlm.nih.gov/pubmed/21068381?dopt=AbstractPlus], http://www.guidetopharmacology.org/GRAC/LigandDisplayForward?ligandId=2644 [http://www.ncbi.nlm.nih.gov/pubmed/21068381?dopt=AbstractPlus]
Comments	Forms a heterodimer with TR4; gene disruption appears without effect on testicular development or function [http://www.ncbi.nlm.nih.gov/pubmed/12052874?dopt=AbstractPlus].	Forms a heterodimer with TR2.

### Further reading on 2C. Testicular receptors

Benoit G *et al*. (2006) International Union of Pharmacology. LXVI. Orphan nuclear receptors. *Pharmacol. Rev*. **58**: 798‐836 https://www.ncbi.nlm.nih.gov/pubmed/17132856?dopt=AbstractPlus

Germain P *et al*. (2006) Overview of nomenclature of nuclear receptors. *Pharmacol. Rev*. **58**: 685‐704 https://www.ncbi.nlm.nih.gov/pubmed/17132848?dopt=AbstractPlus

Safe S *et al*. (2014) Minireview: role of orphan nuclear receptors in cancer and potential as drug targets. *Mol. Endocrinol*. **28**: 157‐72 https://www.ncbi.nlm.nih.gov/pubmed/24295738?dopt=AbstractPlus

Wu D *et al*. (2016) The emerging roles of orphan nuclear receptors in prostate cancer. *Biochim. Biophys. Acta* **1866**: 23‐36 https://www.ncbi.nlm.nih.gov/pubmed/27264242?dopt=AbstractPlus

## http://www.guidetopharmacology.org/GRAC/FamilyDisplayForward?familyId=94

### Overview

Tailless‐like receptors (**nomenclature as agreed by the NC‐IUPHAR Subcommittee on Nuclear Hormone Receptors [** http://www.ncbi.nlm.nih.gov/pubmed/17132856?dopt=AbstractPlus **]**) have yet to be officially paired with an endogenous ligand.

**Table:** 

Nomenclature	http://www.guidetopharmacology.org/GRAC/ObjectDisplayForward?objectId=615	http://www.guidetopharmacology.org/GRAC/ObjectDisplayForward?objectId=616
Systematic nomenclature	NR2E1	NR2E3
HGNC, UniProt	https://www.genenames.org/data/gene‐symbol‐report/#!/hgnc_id/HGNC:7973, http://www.uniprot.org/uniprot/Q9Y466	https://www.genenames.org/data/gene‐symbol‐report/#!/hgnc_id/HGNC:7974, http://www.uniprot.org/uniprot/Q9Y5X4
Comments	Gene disruption is associated with abnormal brain development [http://www.ncbi.nlm.nih.gov/pubmed/12902391?dopt=AbstractPlus, http://www.ncbi.nlm.nih.gov/pubmed/9394001?dopt=AbstractPlus].	–

### Further reading on 2E. Tailless‐like receptors

Benod C *et al*. (2016) TLX: An elusive receptor. *J. Steroid Biochem. Mol. Biol*. **157**: 41‐7 https://www.ncbi.nlm.nih.gov/pubmed/26554934?dopt=AbstractPlus

Benoit G *et al*. (2006) International Union of Pharmacology. LXVI. Orphan nuclear receptors. *Pharmacol. Rev*. **58**: 798‐836 https://www.ncbi.nlm.nih.gov/pubmed/17132856?dopt=AbstractPlus

Germain P *et al*. (2006) Overview of nomenclature of nuclear receptors. *Pharmacol. Rev*. **58**: 685‐704 https://www.ncbi.nlm.nih.gov/pubmed/17132848?dopt=AbstractPlus

O’Leary JD *et al*. (2018) Regulation of behaviour by the nuclear receptor TLX. *Genes Brain Behav*. **17**: e12357 https://www.ncbi.nlm.nih.gov/pubmed/27790850?dopt=AbstractPlus

## http://www.guidetopharmacology.org/GRAC/FamilyDisplayForward?familyId=95

### Overview

COUP‐TF‐like receptors (**nomenclature as agreed by the NC‐IUPHAR Subcommittee on Nuclear Hormone Receptors [** http://www.ncbi.nlm.nih.gov/pubmed/17132856?dopt=AbstractPlus **]**) have yet to be officially paired with an endogenous ligand.

**Table:** 

Nomenclature	http://www.guidetopharmacology.org/GRAC/ObjectDisplayForward?objectId=617	http://www.guidetopharmacology.org/GRAC/ObjectDisplayForward?objectId=618	http://www.guidetopharmacology.org/GRAC/ObjectDisplayForward?objectId=619
Systematic nomenclature	NR2F1	NR2F2	NR2F6
HGNC, UniProt	https://www.genenames.org/data/gene‐symbol‐report/#!/hgnc_id/HGNC:7975, http://www.uniprot.org/uniprot/P10589	https://www.genenames.org/data/gene‐symbol‐report/#!/hgnc_id/HGNC:7976, http://www.uniprot.org/uniprot/P24468	https://www.genenames.org/data/gene‐symbol‐report/#!/hgnc_id/HGNC:7977, http://www.uniprot.org/uniprot/P10588
Comments	Gene disruption is perinatally lethal [http://www.ncbi.nlm.nih.gov/pubmed/9271116?dopt=AbstractPlus].	Gene disruption is embryonically lethal [http://www.ncbi.nlm.nih.gov/pubmed/10215630?dopt=AbstractPlus].	Gene disruption impairs CNS development [http://www.ncbi.nlm.nih.gov/pubmed/15741322?dopt=AbstractPlus].

### Further reading on 2F. COUP‐TF‐like receptors

Benoit G *et al*. (2006) International Union of Pharmacology. LXVI. Orphan nuclear receptors. *Pharmacol. Rev*. **58**: 798‐836 https://www.ncbi.nlm.nih.gov/pubmed/17132856?dopt=AbstractPlus

Germain P *et al*. (2006) Overview of nomenclature of nuclear receptors. *Pharmacol. Rev*. **58**: 685‐704 https://www.ncbi.nlm.nih.gov/pubmed/17132848?dopt=AbstractPlus

Wu D *et al*. (2016) The emerging roles of orphan nuclear receptors in prostate cancer. *Biochim. Biophys. Acta* **1866**: 23‐36 https://www.ncbi.nlm.nih.gov/pubmed/27264242?dopt=AbstractPlus

Wu SP *etal*. (2016) Choose your destiny: Make a cell fate decision with COUP‐TFII. *J. Steroid Biochem. Mol. Biol*. **157**: 7‐12 https://www.ncbi.nlm.nih.gov/pubmed/26658017?dopt=AbstractPlus

## http://www.guidetopharmacology.org/GRAC/FamilyDisplayForward?familyId=97

### Overview

Estrogen‐related receptors (**nomenclature as agreed by the NC‐IUPHAR Subcommittee on Nuclear Hormone Receptors [** http://www.ncbi.nlm.nih.gov/pubmed/17132856?dopt=AbstractPlus **]**) have yet to be officially paired with an endogenous ligand.

**Table:** 

Nomenclature	http://www.guidetopharmacology.org/GRAC/ObjectDisplayForward?objectId=622	http://www.guidetopharmacology.org/GRAC/ObjectDisplayForward?objectId=623	http://www.guidetopharmacology.org/GRAC/ObjectDisplayForward?objectId=624
Systematic nomenclature	NR3B1	NR3B2	NR3B3
HGNC, UniProt	https://www.genenames.org/data/gene‐symbol‐report/#!/hgnc_id/HGNC:3471, http://www.uniprot.org/uniprot/P11474	https://www.genenames.org/data/gene‐symbol‐report/#!/hgnc_id/HGNC:3473, http://www.uniprot.org/uniprot/O95718	https://www.genenames.org/data/gene‐symbol‐report/#!/hgnc_id/HGNC:3474, http://www.uniprot.org/uniprot/P62508
Comments	Activated by some dietary flavonoids [http://www.ncbi.nlm.nih.gov/pubmed/14638870?dopt=AbstractPlus]; activated by the synthetic agonist http://www.guidetopharmacology.org/GRAC/LigandDisplayForward?ligandId=2835 [http://www.ncbi.nlm.nih.gov/pubmed/15857113?dopt=AbstractPlus] and blocked by http://www.guidetopharmacology.org/GRAC/LigandDisplayForward?ligandId=2832 [http://www.ncbi.nlm.nih.gov/pubmed/15184675?dopt=AbstractPlus].	Maybe activated by http://www.guidetopharmacology.org/GRAC/LigandDisplayForward?ligandId=2834 [http://www.ncbi.nlm.nih.gov/pubmed/15713377?dopt=AbstractPlus].	Maybe activated by http://www.guidetopharmacology.org/GRAC/LigandDisplayForward?ligandId=2834 [http://www.ncbi.nlm.nih.gov/pubmed/15713377?dopt=AbstractPlus].

### Further reading on 3B. Estrogen‐related receptors

Benoit G *et al*. (2006) International Union of Pharmacology. LXVI. Orphan nuclear receptors. *Pharmacol. Rev*. **58**: 798‐836 https://www.ncbi.nlm.nih.gov/pubmed/17132856?dopt=AbstractPlus

Divekar SD *et al*. (2016) Estrogen‐related receptor β (ERRβ) ‐ renaissance receptor or receptor renaissance? *Nucl Recept Signal* **14**: e002 https://www.ncbi.nlm.nih.gov/pubmed/27507929?dopt=AbstractPlus

Germain P *et al*. (2006) Overview of nomenclature of nuclear receptors. *Pharmacol. Rev*. **58**: 685‐704 https://www.ncbi.nlm.nih.gov/pubmed/17132848?dopt=AbstractPlus

Tam IS *et al*. (2016) There and back again: The journey of the estrogen‐related receptors in the cancer realm. *J. Steroid Biochem. Mol. Biol*. **157**: 13‐9 https://www.ncbi.nlm.nih.gov/pubmed/26151739?dopt=AbstractPlus

Wu D *et al*. (2016) The emerging roles of orphan nuclear receptors in prostate cancer. *Biochim. Biophys. Acta* **1866**: 23‐36 https://www.ncbi.nlm.nih.gov/pubmed/27264242?dopt=AbstractPlus

## http://www.guidetopharmacology.org/GRAC/FamilyDisplayForward?familyId=99

### Overview

Nerve growth factor IB‐like receptors (**nomenclature as agreed by the NC‐IUPHAR Subcommittee on Nuclear Hormone Receptors [** http://www.ncbi.nlm.nih.gov/pubmed/17132856?dopt=AbstractPlus **]**) have yet to be officially paired with an endogenous ligand.

**Table:** 

Nomenclature	http://www.guidetopharmacology.org/GRAC/ObjectDisplayForward?objectId=629	http://www.guidetopharmacology.org/GRAC/ObjectDisplayForward?objectId=630	http://www.guidetopharmacology.org/GRAC/ObjectDisplayForward?objectId=631
Systematic nomenclature	NR4A1	NR4A2	NR4A3
HGNC, UniProt	https://www.genenames.org/data/gene‐symbol‐report/#!/hgnc_id/HGNC:7980, http://www.uniprot.org/uniprot/P22736	https://www.genenames.org/data/gene‐symbol‐report/#!/hgnc_id/HGNC:7981, http://www.uniprot.org/uniprot/P43354	https://www.genenames.org/data/gene‐symbol‐report/#!/hgnc_id/HGNC:7982, http://www.uniprot.org/uniprot/Q92570
Comments	An endogenous agonist, http://www.guidetopharmacology.org/GRAC/LigandDisplayForward?ligandId=5424, has been described [http://www.ncbi.nlm.nih.gov/pubmed/18690216?dopt=AbstractPlus], although structural analysis and molecular modelling has not identified a ligand binding site [http://www.ncbi.nlm.nih.gov/pubmed/12809604?dopt=AbstractPlus, http://www.ncbi.nlm.nih.gov/pubmed/15716272?dopt=AbstractPlus, http://www.ncbi.nlm.nih.gov/pubmed/12774125?dopt=AbstractPlus].	–	–

### Further reading on 4A. Nerve growth factor IB‐like receptors

Benoit G *et al*. (2006) International Union of Pharmacology. LXVI. Orphan nuclear receptors. *Pharmacol. Rev*. **58**: 798‐836 https://www.ncbi.nlm.nih.gov/pubmed/17132856?dopt=AbstractPlus

Germain P*etal*. (2006) Overview of nomenclature of nuclear receptors. *Pharmacol. Rev*. **58**: 685‐704 https://www.ncbi.nlm.nih.gov/pubmed/17132848?dopt=AbstractPlus

Ranhotra HS. (2015) The NR4A orphan nuclear receptors: mediators in metabolism and diseases. *J. Recept. Signal Transduct. Res*. **35**: 184‐8 https://www.ncbi.nlm.nih.gov/pubmed/25089663?dopt=AbstractPlus

Rodríguez‐Calvo R *et al*. (2017) The NR4A subfamily of nuclear receptors: potential new therapeutic targets for the treatment of inflammatory diseases. *Expert Opin. Ther. Targets* **21**: 291‐304 https://www.ncbi.nlm.nih.gov/pubmed/28055275?dopt=AbstractPlus

Safe S *et al*. (2016) Nuclear receptor 4A (NR4A) family ‐ orphans no more. *J. Steroid Biochem. Mol. Biol*. **157**: 48‐60 https://www.ncbi.nlm.nih.gov/pubmed/25917081?dopt=AbstractPlus

## http://www.guidetopharmacology.org/GRAC/FamilyDisplayForward?familyId=100

### Overview

Fushi tarazu F1‐like receptors (**nomenclature as agreed by the NC‐IUPHAR Subcommittee on Nuclear Hormone Receptors [** http://www.ncbi.nlm.nih.gov/pubmed/17132856?dopt=AbstractPlus **]**) have yet to be officially paired with an endogenous ligand.

**Table:** 

Nomenclature	http://www.guidetopharmacology.org/GRAC/ObjectDisplayForward?objectId=632	http://www.guidetopharmacology.org/GRAC/ObjectDisplayForward?objectId=633
Systematic nomenclature	NR5A1	NR5A2
HGNC, UniProt	https://www.genenames.org/data/gene‐symbol‐report/#!/hgnc_id/HGNC:7983, http://www.uniprot.org/uniprot/Q13285	https://www.genenames.org/data/gene‐symbol‐report/#!/hgnc_id/HGNC:7984, http://www.uniprot.org/uniprot/O00482
Comments	Reported to be inhibited by http://www.guidetopharmacology.org/GRAC/LigandDisplayForward?ligandId=5422 [http://www.ncbi.nlm.nih.gov/pubmed/18055761?dopt=AbstractPlus] and http://www.guidetopharmacology.org/GRAC/LigandDisplayForward?ligandId=5423 [http://www.ncbi.nlm.nih.gov/pubmed/18334597?dopt=AbstractPlus].	–

### Further reading on 5A. Fushi tarazu F1‐like receptors

Benoit G *et al*. (2006) International Union of Pharmacology. LXVI. Orphan nuclear receptors. *Pharmacol. Rev*. **58**: 798‐836 https://www.ncbi.nlm.nih.gov/pubmed/17132856?dopt=AbstractPlus

Garattini E *et al*. (2016) Lipid‐sensors, enigmatic‐orphan and orphan nuclear receptors as therapeutic targets in breast‐cancer. *Oncotarget* **7**: 42661‐42682 https://www.ncbi.nlm.nih.gov/pubmed/26894976?dopt=AbstractPlus

Germain P *et al*. (2006) Overview of nomenclature of nuclear receptors. *Pharmacol. Rev*. **58**: 685‐704 https://www.ncbi.nlm.nih.gov/pubmed/17132848?dopt=AbstractPlus

Zhi X *et al*. (2016) Structures and regulation of non‐X orphan nuclear receptors: A retinoid hypothesis. *J. Steroid Biochem. Mol. Biol*. **157**: 27‐40 https://www.ncbi.nlm.nih.gov/pubmed/26159912?dopt=AbstractPlus

Zimmer V *et al*. (2015) Nuclear receptor variants in liver disease. *Dig Dis* **33**: 415‐9 https://www.ncbi.nlm.nih.gov/pubmed/26045277?dopt=AbstractPlus

## http://www.guidetopharmacology.org/GRAC/FamilyDisplayForward?familyId=101

### Overview

Germ cell nuclear factor receptors (**nomenclature as agreed by the NC‐IUPHAR Subcommittee on Nuclear Hormone Receptors [** http://www.ncbi.nlm.nih.gov/pubmed/17132856?dopt=AbstractPlus **]**) have yet to be officially paired with an endogenous ligand.

**Table:** 

Nomenclature	http://www.guidetopharmacology.org/GRAC/ObjectDisplayForward?objectId=634
Systematic nomenclature	NR6A1
HGNC, UniProt	https://www.genenames.org/data/gene‐symbol‐report/#!/hgnc_id/HGNC:7985, http://www.uniprot.org/uniprot/Q15406

### Further reading on 6A. Germ cell nuclear factor receptors

Benoit G *et al*. (2006) International Union of Pharmacology. LXVI. Orphan nuclear receptors. *Pharmacol. Rev*. **58**: 798‐836 https://www.ncbi.nlm.nih.gov/pubmed/17132856?dopt=AbstractPlus

Garattini E *et al*. (2016) Lipid‐sensors, enigmatic‐orphan and orphan nuclear receptors as therapeutic in breast‐cancer. *Oncotarget* **7**: 42661‐42682 https://www.ncbi.nlm.nih.gov/pubmed/26894976?dopt=AbstractPlus

Germain P *et al*. (2006) Overview of nomenclature of nuclear receptors. *Pharmacol. Rev*. **58**: 685‐704 https://www.ncbi.nlm.nih.gov/pubmed/17132848?dopt=AbstractPlus

Safe S *et al*. (2014) Minireview: role of orphan nuclear receptors in cancer and potential as drug *Mol. Endocrinol*. **28**: 157‐72 https://www.ncbi.nlm.nih.gov/pubmed/24295738?dopt=AbstractPlus

Zhi X *et al*. (2016) Structures and regulation of non‐X orphan nuclear receptors: A retinoid hypothesis. *J. Steroid Biochem. Mol. Biol*. **157**: 27‐40 https://www.ncbi.nlm.nih.gov/pubmed/26159912?dopt=AbstractPlus

## http://www.guidetopharmacology.org/GRAC/FamilyDisplayForward?familyId=102

### Overview

Dax‐like receptors (**nomenclature as agreed by the** NC‐IUPHAR **Subcommittee on Nuclear Hormone Receptors [** http://www.ncbi.nlm.nih.gov/pubmed/17132856?dopt=AbstractPlus **]**) have yet to be officially paired with an endogenous ligand.

**Table:** 

Nomenclature	http://www.guidetopharmacology.org/GRAC/ObjectDisplayForward?objectId=635	http://www.guidetopharmacology.org/GRAC/ObjectDisplayForward?objectId=636
Systematic nomenclature	NR0B1	NR0B2
HGNC, UniProt	https://www.genenames.org/data/gene‐symbol‐report/#!/hgnc_id/HGNC:7960, http://www.uniprot.org/uniprot/P51843	https://www.genenames.org/data/gene‐symbol‐report/#!/hgnc_id/HGNC:7961, http://www.uniprot.org/uniprot/Q15466

### Further reading on 0B. DAX‐like receptors

Benoit G *et al*. (2006) International Union of Pharmacology. LXVI. Orphan nuclear receptors. *Pharmacol. Rev*. **58**: 798‐836 https://www.ncbi.nlm.nih.gov/pubmed/17132856?dopt=AbstractPlus

Garattini E *et al*. (2016) Lipid‐sensors, enigmatic‐orphan and orphan nuclear receptors as therapeutic targets in breast‐cancer. *Oncotarget* **7**: 42661‐42682 https://www.ncbi.nlm.nih.gov/pubmed/26894976?dopt=AbstractPlus

Germain P *et al*. (2006) Overview of nomenclature of nuclear receptors. *Pharmacol. Rev*. **58**: 685‐704 https://www.ncbi.nlm.nih.gov/pubmed/17132848?dopt=AbstractPlus

Safe S *et al*. (2014) Minireview: role of orphan nuclear receptors in cancer and potential as drug targets. *Mol. Endocrinol*. **28**: 157‐72 https://www.ncbi.nlm.nih.gov/pubmed/24295738?dopt=AbstractPlus

Wu D *et al*. (2016) The emerging roles of orphan nuclear receptors in prostate cancer. *Biochim. Biophys. Acta* **1866**: 23‐36 https://www.ncbi.nlm.nih.gov/pubmed/27264242?dopt=AbstractPlus

## http://www.guidetopharmacology.org/GRAC/FamilyDisplayForward?familyId=107

### Overview

Steroid hormone receptors (**nomenclature as agreed by the NC‐IUPHAR Subcommittee on Nuclear Hormone Receptors [** http://www.ncbi.nlm.nih.gov/pubmed/17132854?dopt=AbstractPlus, http://www.ncbi.nlm.nih.gov/pubmed/17132855?dopt=AbstractPlus **]**) are nuclear hormone receptors of the NR3 class, with endogenous agonists that may be divided into 3‐hydroxysteroids (http://www.guidetopharmacology.org/GRAC/LigandDisplayForward?ligandId=2818 and http://www.guidetopharmacology.org/GRAC/LigandDisplayForward?ligandId=1013) and 3‐ketosteroids (http://www.guidetopharmacology.org/GRAC/LigandDisplayForward?ligandId=2856 [DHT], http://www.guidetopharmacology.org/GRAC/LigandDisplayForward?ligandId=2872, http://www.guidetopharmacology.org/GRAC/LigandDisplayForward?ligandId=2868, http://www.guidetopharmacology.org/GRAC/LigandDisplayForward?ligandId=2869, http://www.guidetopharmacology.org/GRAC/LigandDisplayForward?ligandId=2377 and http://www.guidetopharmacology.org/GRAC/LigandDisplayForward?ligandId=2858). These receptors exist as dimers coupled with chaperone molecules (such as http://www.guidetopharmacology.org/GRAC/LigandDisplayForward?ligandId=5365 (https://www.genenames.org/data/gene‐symbol‐report/#!/hgnc_id/HGNC:5258, http://www.uniprot.org/uniprot/P08238) and immunophilin FKBP52:https://www.genenames.org/data/gene‐symbol‐report/#!/hgnc_id/HGNC:3720, http://www.uniprot.org/uniprot/Q02790), which are shed on binding the steroid hormone. Although rapid signalling phenomena are observed [http://www.ncbi.nlm.nih.gov/pubmed/18784332?dopt=AbstractPlus, http://www.ncbi.nlm.nih.gov/pubmed/19389460?dopt=AbstractPlus], the principal signalling cascade appears to involve binding of the activated receptors to nuclear hormone response elements of the genome, with a 15‐nucleotide consensus sequence AGAACAnnnTGTTCT (*i.e*. an inverted palindrome) as homo‐ or heterodimers. They also affect transcription by protein‐protein interactions with other transcription factors, such as activator protein 1 (AP‐1) and nuclear factor *_κ_*B (NF‐*_κ_*B). Splice variants of each of these receptors can form functional or non‐functional monomers that can dimerize to form functional or non‐functional receptors. For example, alternative splicing of PR mRNA produces A and B monomers that combine to produce functional AA, AB and BB receptors with distinct characteristics [http://www.ncbi.nlm.nih.gov/pubmed/8264658?dopt=AbstractPlus].

A 7TM receptor responsive to estrogen (https://www.genenames.org/data/gene‐symbol‐report/#!/hgnc_id/HGNC:4485, http://www.uniprot.org/uniprot/Q99527, also known as GPR30, see [http://www.ncbi.nlm.nih.gov/pubmed/18271749?dopt=AbstractPlus]) has been described. Human orthologues of 7TM ’membrane progestin receptors’ (https://www.genenames.org/data/gene‐symbol‐report/#!/hgnc_id/HGNC:23146, https://www.genenames.org/data/gene‐symbol‐report/#!/hgnc_id/HGNC:15708 and https://www.genenames.org/data/gene‐symbol‐report/#!/hgnc_id/HGNC:29645), initially discovered in fish [http://www.ncbi.nlm.nih.gov/pubmed/12601167?dopt=AbstractPlus, http://www.ncbi.nlm.nih.gov/pubmed/12574519?dopt=AbstractPlus], appear to localize to intracellular membranes and respond to ’non‐genomic’ progesterone analogues independently of G proteins [http://www.ncbi.nlm.nih.gov/pubmed/18603275?dopt=AbstractPlus].

## http://www.guidetopharmacology.org/GRAC/FamilyDisplayForward?familyId=96

### Overview

Estrogen receptor (ER) activity regulates diverse physiological processes *via* transcriptional modulation of target genes. The selection of target genes and the magnitude of the response, be it induction or repression, are determined by many factors, including the effect of the hormone ligand and DNA binding on ER structural conformation, and the local cellular regulatory environment. The cellular environment defines the specific complement of DNA enhancer and promoter elements present and the availability of coregulators to form functional transcription complexes. Together, these determinants control the resulting biological response.

**Table:** 

Nomenclature	http://www.guidetopharmacology.org/GRAC/ObjectDisplayForward?objectId=620	http://www.guidetopharmacology.org/GRAC/ObjectDisplayForward?objectId=621
Systematic nomenclature	NR3A1	NR3A2
HGNC, UniProt	https://www.genenames.org/data/gene‐symbol‐report/#!/hgnc_id/HGNC:3467, http://www.uniprot.org/uniprot/P03372	https://www.genenames.org/data/gene‐symbol‐report/#!/hgnc_id/HGNC:3468, http://www.uniprot.org/uniprot/Q92731
Endogenous agonists	http://www.guidetopharmacology.org/GRAC/LigandDisplayForward?ligandId=2821 [http://www.ncbi.nlm.nih.gov/pubmed/9048584?dopt=AbstractPlus], http://www.guidetopharmacology.org/GRAC/LigandDisplayForward?ligandId=2818 [http://www.ncbi.nlm.nih.gov/pubmed/9048584?dopt=AbstractPlus]	–
Selective agonists	http://www.guidetopharmacology.org/GRAC/LigandDisplayForward?ligandId=2819 [http://www.ncbi.nlm.nih.gov/pubmed/11014206?dopt=AbstractPlus, http://www.ncbi.nlm.nih.gov/pubmed/11150164?dopt=AbstractPlus], http://www.guidetopharmacology.org/GRAC/LigandDisplayForward?ligandId=7071 [http://www.ncbi.nlm.nih.gov/pubmed/16722623?dopt=AbstractPlus]	http://www.guidetopharmacology.org/GRAC/LigandDisplayForward?ligandId=4052 [http://www.ncbi.nlm.nih.gov/pubmed/15456246?dopt=AbstractPlus], http://www.guidetopharmacology.org/GRAC/LigandDisplayForward?ligandId=2825 [http://www.ncbi.nlm.nih.gov/pubmed/11708925?dopt=AbstractPlus, http://www.ncbi.nlm.nih.gov/pubmed/11150164?dopt=AbstractPlus], http://www.guidetopharmacology.org/GRAC/LigandDisplayForward?ligandId=6700 [http://www.ncbi.nlm.nih.gov/pubmed/18097065?dopt=AbstractPlus, http://www.ncbi.nlm.nih.gov/pubmed/15456246?dopt=AbstractPlus]
Sub/family‐selective antagonists	http://www.guidetopharmacology.org/GRAC/LigandDisplayForward?ligandId=7355 (pIC_50_ 7.6) [http://www.ncbi.nlm.nih.gov/pubmed/11356100?dopt=AbstractPlus]	http://www.guidetopharmacology.org/GRAC/LigandDisplayForward?ligandId=7355 (pIC_50_ 7.1) [http://www.ncbi.nlm.nih.gov/pubmed/11356100?dopt=AbstractPlus]
Selective antagonists	http://www.guidetopharmacology.org/GRAC/LigandDisplayForward?ligandId=4159 (p*K*_i_ 8.9) [2], http://www.guidetopharmacology.org/GRAC/LigandDisplayForward?ligandId=3459 (p*K*_i_ 8.6) [http://www.ncbi.nlm.nih.gov/pubmed/11861516?dopt=AbstractPlus]	http://www.guidetopharmacology.org/GRAC/LigandDisplayForward?ligandId=2822 (p*K*_i_ 8.4) [http://www.ncbi.nlm.nih.gov/pubmed/10395487?dopt=AbstractPlus, http://www.ncbi.nlm.nih.gov/pubmed/9927308?dopt=AbstractPlus], http://www.guidetopharmacology.org/GRAC/LigandDisplayForward?ligandId=3461 (p*K*_i_ 6.9) [http://www.ncbi.nlm.nih.gov/pubmed/17228884?dopt=AbstractPlus]

### Comments

http://www.guidetopharmacology.org/GRAC/LigandDisplayForward?ligandId=2822 exhibits partial agonist activity at ERα [http://www.ncbi.nlm.nih.gov/pubmed/10395487?dopt=AbstractPlus, http://www.ncbi.nlm.nih.gov/pubmed/9927308?dopt=AbstractPlus]. Estrogen receptors may be blocked non‐selectively by http://www.guidetopharmacology.org/GRAC/LigandDisplayForward?ligandId=1016 and http://www.guidetopharmacology.org/GRAC/LigandDisplayForward?ligandId=2820 and labelled by http://www.guidetopharmacology.org/GRAC/LigandDisplayForward?ligandId=1012 and http://www.guidetopharmacology.org/GRAC/LigandDisplayForward?ligandId=5384. Many agents thought initially to be antagonists at estrogen receptors appear to have tissue‐specific efficacy (*e.g*. http://www.guidetopharmacology.org/GRAC/LigandDisplayForward?ligandId=1016 is an antagonist at estrogen receptors in the breast, but is an agonist at estrogen receptors in the uterus), hence the descriptor SERM (selective estrogen receptor modulator) or SnuRM (selective nuclear receptor modulator). http://www.guidetopharmacology.org/GRAC/LigandDisplayForward?ligandId=5430 has been suggested to be an ERα‐selective estrogen receptor modulator [http://www.ncbi.nlm.nih.gov/pubmed/17115070?dopt=AbstractPlus].

### Further reading on 3A. Estrogen receptors

Coons LA *et al*. (2017) DNA Sequence Constraints Define Functionally Active Steroid Nuclear Receptor Binding Sites in Chromatin. *Endocrinology* **158**: 3212‐3234 https://www.ncbi.nlm.nih.gov/pubmed/28977594?dopt=AbstractPlus

Dahlman‐Wright K *et al*. (2006) International Union of Pharmacology. LXIV. Estrogen receptors. *Pharmacol. Rev*. **58**: 773‐81 https://www.ncbi.nlm.nih.gov/pubmed/17132854?dopt=AbstractPlus

Gonzalez‐Sanchez E *et al*. (2015) Nuclear receptors in acute and chronic cholestasis. *Dig Dis* **33**: 357‐66 https://www.ncbi.nlm.nih.gov/pubmed/26045270?dopt=AbstractPlus

Hewitt SC *et al*. (2016) What’s new in estrogen receptor action in the female reproductive tract. *J. Mol. Endocrinol*. **56**: R55‐71 https://www.ncbi.nlm.nih.gov/pubmed/26826253?dopt=AbstractPlus

Jameera Begam A *et al*. (2017) Estrogen receptor agonists/antagonists in breast cancer therapy: A critical review. *Bioorg. Chem*. **71**: 257‐274 https://www.ncbi.nlm.nih.gov/pubmed/28274582?dopt=AbstractPlus

Warner M *et al*. (2017) Estrogen Receptor β as a Pharmaceutical Target. *Trends Pharmacol. Sci*. **38**: 92‐99 https://www.ncbi.nlm.nih.gov/pubmed/27979317?dopt=AbstractPlus

## http://www.guidetopharmacology.org/GRAC/FamilyDisplayForward?familyId=98

### Overview

Steroid hormone receptors (**nomenclature as agreed by the NC‐IUPHAR Subcommittee on Nuclear Hormone Receptors [** http://www.ncbi.nlm.nih.gov/pubmed/17132854?dopt=AbstractPlus, https://www.ncbi.nlm.nih.gov/pubmed/17132855?dopt=AbstractPlus **]**) are nuclear hormone receptors of the NR3 class, with endogenous agonists that may be divided into 3‐hydroxysteroids (http://www.guidetopharmacology.org/GRAC/LigandDisplayForward?ligandId=2818 and http://www.guidetopharmacology.org/GRAC/LigandDisplayForward?ligandId=1013) and 3‐ketosteroids (http://www.guidetopharmacology.org/GRAC/LigandDisplayForward?ligandId=2856 [DHT], http://www.guidetopharmacology.org/GRAC/LigandDisplayForward?ligandId=2872, http://www.guidetopharmacology.org/GRAC/LigandDisplayForward?ligandId=2868, http://www.guidetopharmacology.org/GRAC/LigandDisplayForward?ligandId=2869, http://www.guidetopharmacology.org/GRAC/LigandDisplayForward?ligandId=2377 and http://www.guidetopharmacology.org/GRAC/LigandDisplayForward?ligandId=2858).

**Table:** 

Nomenclature	http://www.guidetopharmacology.org/GRAC/ObjectDisplayForward?objectId=628	http://www.guidetopharmacology.org/GRAC/ObjectDisplayForward?objectId=625	http://www.guidetopharmacology.org/GRAC/ObjectDisplayForward?objectId=626	http://www.guidetopharmacology.org/GRAC/ObjectDisplayForward?objectId=627
Systematic nomenclature	NR3C4	NR3C1	NR3C2	NR3C3
HGNC, UniProt	https://www.genenames.org/data/gene‐symbol‐report/#!/hgnc_id/HGNC:644, http://www.uniprot.org/uniprot/P10275	https://www.genenames.org/data/gene‐symbol‐report/#!/hgnc_id/HGNC:7978, http://www.uniprot.org/uniprot/P04150	https://www.genenames.org/data/gene‐symbol‐report/#!/hgnc_id/HGNC:7979, http://www.uniprot.org/uniprot/P08235	https://www.genenames.org/data/gene‐symbol‐report/#!/hgnc_id/HGNC:8910, http://www.uniprot.org/uniprot/P06401
Rank order of potency	http://www.guidetopharmacology.org/GRAC/LigandDisplayForward?ligandId=2856 *>* http://www.guidetopharmacology.org/GRAC/LigandDisplayForward?ligandId=2858	http://www.guidetopharmacology.org/GRAC/LigandDisplayForward?ligandId=2868, http://www.guidetopharmacology.org/GRAC/LigandDisplayForward?ligandId=2869 ≫ http://www.guidetopharmacology.org/GRAC/LigandDisplayForward?ligandId=2872, http://www.guidetopharmacology.org/GRAC/LigandDisplayForward?ligandId=3450 [http://www.ncbi.nlm.nih.gov/pubmed/8282004?dopt=AbstractPlus]	http://www.guidetopharmacology.org/GRAC/LigandDisplayForward?ligandId=2869, http://www.guidetopharmacology.org/GRAC/LigandDisplayForward?ligandId=2868, http://www.guidetopharmacology.org/GRAC/LigandDisplayForward?ligandId=2872, http://www.guidetopharmacology.org/GRAC/LigandDisplayForward?ligandId=2377 [http://www.ncbi.nlm.nih.gov/pubmed/8282004?dopt=AbstractPlus]	http://www.guidetopharmacology.org/GRAC/LigandDisplayForward?ligandId=2377
Endogenous agonists	http://www.guidetopharmacology.org/GRAC/LigandDisplayForward?ligandId=2856 [http://www.ncbi.nlm.nih.gov/pubmed/2911578?dopt=AbstractPlus]	–	http://www.guidetopharmacology.org/GRAC/LigandDisplayForward?ligandId=2872 [http://www.ncbi.nlm.nih.gov/pubmed/10611474?dopt=AbstractPlus, http://www.ncbi.nlm.nih.gov/pubmed/8282004?dopt=AbstractPlus]	http://www.guidetopharmacology.org/GRAC/LigandDisplayForward?ligandId=2377 [http://www.ncbi.nlm.nih.gov/pubmed/9667968?dopt=AbstractPlus]
Selective agonists	http://www.guidetopharmacology.org/GRAC/LigandDisplayForward?ligandId=7100 [http://www.ncbi.nlm.nih.gov/pubmed/17574413?dopt=AbstractPlus], http://www.guidetopharmacology.org/GRAC/LigandDisplayForward?ligandId=2859 [http://www.ncbi.nlm.nih.gov/pubmed/10852459?dopt=AbstractPlus], http://www.guidetopharmacology.org/GRAC/LigandDisplayForward?ligandId=2861 [http://www.ncbi.nlm.nih.gov/pubmed/17439112?dopt=AbstractPlus], http://www.guidetopharmacology.org/GRAC/LigandDisplayForward?ligandId=2857 [http://www.ncbi.nlm.nih.gov/pubmed/10076535?dopt=AbstractPlus], http://www.guidetopharmacology.org/GRAC/LigandDisplayForward?ligandId=6947	http://www.guidetopharmacology.org/GRAC/LigandDisplayForward?ligandId=7080 [http://www.ncbi.nlm.nih.gov/pubmed/10633034?dopt=AbstractPlus], http://www.guidetopharmacology.org/GRAC/LigandDisplayForward?ligandId=7076 [2], http://www.guidetopharmacology.org/GRAC/LigandDisplayForward?ligandId=7059 [2], http://www.guidetopharmacology.org/GRAC/LigandDisplayForward?ligandId=7088 [2], http://www.guidetopharmacology.org/GRAC/LigandDisplayForward?ligandId=7061 [2], http://www.guidetopharmacology.org/GRAC/LigandDisplayForward?ligandId=7434 [http://www.ncbi.nlm.nih.gov/pubmed/21880489?dopt=AbstractPlus]	–	http://www.guidetopharmacology.org/GRAC/LigandDisplayForward?ligandId=2879 (Affinity at human PR‐A) [http://www.ncbi.nlm.nih.gov/pubmed/9464360?dopt=AbstractPlus], http://www.guidetopharmacology.org/GRAC/LigandDisplayForward?ligandId=3453, http://www.guidetopharmacology.org/GRAC/LigandDisplayForward?ligandId=2881 [http://www.ncbi.nlm.nih.gov/pubmed/6645495?dopt=AbstractPlus, http://www.ncbi.nlm.nih.gov/pubmed/14670641?dopt=AbstractPlus]
Selective antagonists	http://www.guidetopharmacology.org/GRAC/LigandDisplayForward?ligandId=2863 (p*K*_i_ 7.7) [http://www.ncbi.nlm.nih.gov/pubmed/16420057?dopt=AbstractPlus], http://www.guidetopharmacology.org/GRAC/LigandDisplayForward?ligandId=6698 (pIC_50_ 7.1–7.5) [http://www.ncbi.nlm.nih.gov/pubmed/18921992?dopt=AbstractPlus], http://www.guidetopharmacology.org/GRAC/LigandDisplayForward?ligandId=6812 (pIC_50_ 7.4) [http://www.ncbi.nlm.nih.gov/pubmed/19359544?dopt=AbstractPlus], http://www.guidetopharmacology.org/GRAC/LigandDisplayForward?ligandId=2864 (pIC_50_ 7.1–7.1) [http://www.ncbi.nlm.nih.gov/pubmed/9111629?dopt=AbstractPlus], http://www.guidetopharmacology.org/GRAC/LigandDisplayForward?ligandId=2862 (pEC_50_ 6.6) [http://www.ncbi.nlm.nih.gov/pubmed/10076535?dopt=AbstractPlus], http://www.guidetopharmacology.org/GRAC/LigandDisplayForward?ligandId=8638 (pIC_50_ 6.4) [http://www.ncbi.nlm.nih.gov/pubmed/15828836?dopt=AbstractPlus], http://www.guidetopharmacology.org/GRAC/LigandDisplayForward?ligandId=6943 (Displacement of ^3^[H] testosterone from wild‐type androgen receptors) (p*K*_i_ 5.4) [http://www.ncbi.nlm.nih.gov/pubmed/18571420?dopt=AbstractPlus]	http://www.guidetopharmacology.org/GRAC/LigandDisplayForward?ligandId=2882 (pIC_50_ 7.6) [http://www.ncbi.nlm.nih.gov/pubmed/12781198?dopt=AbstractPlus], http://www.guidetopharmacology.org/GRAC/LigandDisplayForward?ligandId=3448	http://www.guidetopharmacology.org/GRAC/LigandDisplayForward?ligandId=8678 (pIC_50_ 7.7) [http://www.ncbi.nlm.nih.gov/pubmed/22791416?dopt=AbstractPlus], http://www.guidetopharmacology.org/GRAC/LigandDisplayForward?ligandId=2876 (p*K*_i_ 6.9) [http://www.ncbi.nlm.nih.gov/pubmed/18038968?dopt=AbstractPlus], http://www.guidetopharmacology.org/GRAC/LigandDisplayForward?ligandId=2882 (pIC_50_ 6.3) [http://www.ncbi.nlm.nih.gov/pubmed/12781198?dopt=AbstractPlus], http://www.guidetopharmacology.org/GRAC/LigandDisplayForward?ligandId=3451, http://www.guidetopharmacology.org/GRAC/LigandDisplayForward?ligandId=3448	http://www.guidetopharmacology.org/GRAC/LigandDisplayForward?ligandId=7460 (pIC_50_ 9.7) [http://www.ncbi.nlm.nih.gov/pubmed/18243712?dopt=AbstractPlus], http://www.guidetopharmacology.org/GRAC/LigandDisplayForward?ligandId=2805 (Mixed) (p*K*_i_ 9) [http://www.ncbi.nlm.nih.gov/pubmed/12781197?dopt=AbstractPlus], http://www.guidetopharmacology.org/GRAC/LigandDisplayForward?ligandId=2882 (p*K*_i_ 7.7) [http://www.ncbi.nlm.nih.gov/pubmed/8627601?dopt=AbstractPlus], http://www.guidetopharmacology.org/GRAC/LigandDisplayForward?ligandId=3448
Labelled ligands	http://www.guidetopharmacology.org/GRAC/LigandDisplayForward?ligandId=3455 (Selective Agonist), http://www.guidetopharmacology.org/GRAC/LigandDisplayForward?ligandId=3457 (Selective Agonist), http://www.guidetopharmacology.org/GRAC/LigandDisplayForward?ligandId=3456 (Agonist)	http://www.guidetopharmacology.org/GRAC/LigandDisplayForward?ligandId=3447 (Agonist)	http://www.guidetopharmacology.org/GRAC/LigandDisplayForward?ligandId=3452 (Selective Agonist) [http://www.ncbi.nlm.nih.gov/pubmed/16188378?dopt=AbstractPlus, http://www.ncbi.nlm.nih.gov/pubmed/6320679?dopt=AbstractPlus] – Rat	http://www.guidetopharmacology.org/GRAC/LigandDisplayForward?ligandId=3454 (Selective Agonist)

### Comments

http://www.guidetopharmacology.org/GRAC/LigandDisplayForward?ligandId=3447 also binds to MR*in vitro*. PR antagonists have been suggested to subdivide into Type I (*e.g*. http://www.guidetopharmacology.org/GRAC/LigandDisplayForward?ligandId=2882) and Type II (*e.g*. http://www.guidetopharmacology.org/GRAC/LigandDisplayForward?ligandId=3448) groups. These groups appear to promote binding of PR to DNA with different efficacies and evoke distinct conformational changes in the receptor, leading to a transcription‐neutral complex [http://www.ncbi.nlm.nih.gov/pubmed/9528977?dopt=AbstractPlus, http://www.ncbi.nlm.nih.gov/pubmed/9849965?dopt=AbstractPlus]. Mutations in AR underlie testicular feminization and androgen insensitivity syndromes, spinal and bulbar muscular atrophy (Kennedy’s disease).

### Further reading on 3C. 3‐Ketosteroid receptors

Baker ME *et al*. (2017) 30 YEARS OF THE MINERALOCORTICOID RECEPTOR: Evolution of the mineralocorticoid receptor: sequence, structure and function. *J. Endocrinol*. **234**: T1‐T16 https://www.ncbi.nlm.nih.gov/pubmed/28468932?dopt=AbstractPlus

Carroll JS *et al*. (2017) Deciphering the divergent roles of progestogens in breast cancer. *Nat. Rev. Cancer* **17**: 54‐64 https://www.ncbi.nlm.nih.gov/pubmed/27885264?dopt=AbstractPlus

Cohen DM *et al*. (2017) Nuclear Receptor Function through Genomics: Lessons from the Glucocorticoid Receptor. *Trends Endocrinol. Metab*. **28**: 531‐540 https://www.ncbi.nlm.nih.gov/pubmed/28495406?dopt=AbstractPlus

de Kloet ER *et al*. (2017) Brain mineralocorticoid receptor function in control of salt balance and stress‐adaptation. *Physiol. Behav*. **178**: 13‐20 https://www.ncbi.nlm.nih.gov/pubmed/28089704?dopt=AbstractPlus

Garg D *et al*. (2017) Progesterone‐Mediated Non‐Classical Signaling. *Trends Endocrinol. Metab*. **28**: 656‐668 https://www.ncbi.nlm.nih.gov/pubmed/28651856?dopt=AbstractPlus

Lu NZ *et al*. (2006) International Union of Pharmacology. LXV. The pharmacology and classification of the nuclear receptor superfamily: glucocorticoid, mineralocorticoid, progesterone, and androgen receptors. *Pharmacol. Rev*. **58**: 782‐97 https://www.ncbi.nlm.nih.gov/pubmed/17132855?dopt=AbstractPlus

Lucas‐Herald AK *et al*. (2017) Genomic and non‐genomic effects of androgens in the cardiovascular system: clinical implications. *Clin. Sci*. **131**: 1405‐1418 https://www.ncbi.nlm.nih.gov/pubmed/28645930?dopt=AbstractPlus

Wadosky KM *et al*. (2017) Androgen receptor splice variants and prostate cancer: From bench to bedside. *Oncotarget* **8**: 18550‐18576 https://www.ncbi.nlm.nih.gov/pubmed/28077788?dopt=AbstractPlus

Weikum ER *et al*. (2017) Glucocorticoid receptor control of transcription: precision and plasticity via allostery. *Nat. Rev. Mol. Cell Biol*. **18**: 159‐174 https://www.ncbi.nlm.nih.gov/pubmed/28053348?dopt=AbstractPlus
